# Restoration of axon initial segment plasticity via chemogenetic activation rescues autism-related behaviors

**DOI:** 10.1038/s41419-026-08873-0

**Published:** 2026-05-19

**Authors:** Yoshinori Otani, Xiaowei Zhu, Xinlang Liu, Kohei Koga, Ryo Kawabata, Hisao Miyajima, Toru Takumi, Masashi Fujitani

**Affiliations:** 1 Department of Anatomy and Neuroscience, Faculty of Medicine, Shimane University, Izumo, Shimane Japan; 2 Department of Neurophysiology, Faculty of Medicine, Hyogo Medical University, Nishinomiya, Hyogo Japan; 3 Gladstone Institute of Neurological Disease, San Francisco, CA USA; 4 Department of Physiology and Cell Biology, Kobe University School of Medicine, Chuo-ku, Kobe, Hyogo Japan

**Keywords:** Cellular neuroscience, Brain, Molecular neuroscience

## Abstract

Autism spectrum disorder (ASD) presents a major clinical challenge, necessitating the identification of novel therapeutic targets rooted in its underlying pathophysiology. The axon initial segment (AIS) is the critical site for action potential initiation and a hub for homeostatic plasticity; however, its involvement in ASD remains poorly defined. Herein, we report significant structural and functional deficits in the AIS within a clinically relevant ASD mouse model harboring a *15q11-13 duplication* (*15q dup*). We observed that pyramidal neurons in the medial prefrontal cortex (mPFC) exhibited shortened AIS, resulting in reduced neuronal excitability and impaired plasticity. Importantly, these abnormalities were specific to long-range circuits, including the mPFC–dorsal raphe nucleus (DRN) pathway, which is critical for social behavior. We employed a circuit-specific chemogenetic strategy that activates these mPFC–DRN projection neurons to test the reversibility of this phenotype. Remarkably, this targeted intervention normalized AIS structure and rescued core ASD-like behaviors, including social interaction deficits and repetitive behaviors. These results demonstrated that AIS alterations in this ASD model represent a reversible form of maladaptive plasticity, rather than permanent neuropathology. Our study highlights circuit-specific AIS modulation as a promising novel avenue for therapeutic interventions aimed at correcting fundamental neuronal excitability deficits in ASD.

## Introduction

The axon initial segment (AIS), located in the proximal region of the axon, is characterized by a high density of the anchoring protein Ankyrin-G and various ion channels, which together play a pivotal role in initiating action potentials (APs) [1, 2]. AIS serves as a critical regulator of neuronal excitability and is the primary site of AP initiation [2–6]. Alterations in axonal structural features, such as length and position, influence neuronal function [2–6]. This activity-dependent AIS plasticity [2, 7, 8] has been demonstrated by in vivo studies utilizing sensory deprivation paradigms such as tactile [9], auditory [10, 11], visual [12, 13], and olfactory deprivation [14].

Emerging research has linked AIS abnormalities to a spectrum of neurological disorders [2]. Specifically, increased cellular excitability with changes in AIS plasticity has been observed in animal models of Fragile X syndrome [15]. Ankyrin-G protein expression and AIS length were altered and accompanied by neuronal hyperexcitability in animal models of Angelman syndrome [16]. Moreover, *Tau* mutations causing frontotemporal dementia (FTD) abolish AIS plasticity and impair neuronal activity through AIS cytoskeletal changes induced by end-binding protein 3 (EB3) [17]. These findings suggest that AIS alterations occur under both physiological and pathological conditions, but raise a critical question: do these changes reflect irreversible neuropathology, as seen in FTD, or a reversible adaptive state?

Autism spectrum disorder (ASD) is a complex developmental disorder marked by deficits in social communication, restricted interests, repetitive behaviors, and variable intellectual disability [18, 19]. Copy number variations (CNVs) are chromosomal abnormalities that represent significant risk factors for the disorder [20]. CNVs within the *15q11–13* locus have been identified as genomic variants associated with ASD [21]. Moreover, mouse models with duplication of the syntenic human *15q11–13* region (*15q dup*) have been developed and exhibit ASD-like symptoms [22], increased somatosensory cortex activity, and decreased 5-hydroxytryptamine (5-HT) neuronal activity, reflecting abnormal excitatory/inhibitory (E/I) balance [23].

To understand how brain-wide activity patterns contribute to ASD-like behaviors, previous studies have elucidated global and neural circuit dysfunctions in *15q dup* ASD mouse models using awake functional magnetic resonance imaging [24] and real-time imaging [25]. While these studies highlighted abnormal network activity, aberrant plasticity emerged as a key factor in ASD pathophysiology. Although abnormal synaptic plasticity has been extensively studied in ASD [26, 27], how AIS plasticity contributes to the E/I imbalance or whether these AIS abnormalities are reversible remains unclear. We hypothesized that AIS shortening in *15q dup* mice represents a reversible form of maladaptive plasticity. Therefore, this study sought to determine whether restoring normal activity within specific neural circuits could rescue the AIS structure and associated behavioral deficits.

## Materials and methods

### Mice

*15q dup* mice were generated using a chromosome engineering technique [22] and maintained on a C57BL/6J background. B6.FVB(Cg)-Tg(*Rbp4-Cre*)KL100Gsat/Mmucd (RRID: MMRRC_037128-UCD) (*Rbp4-Cre* mice) were obtained from Mutant Mouse Resource & Research Centers.

Genotyping of mouse tail genomic DNA by polymerase chain reaction (PCR) was performed as previously described, with slight modifications [28]. PCR was performed using GoTaq Green Master Mix (Promega, Madison, WI, USA) with the following primer sets: For *15q dup* mice: hprt-intron2-F1: AGAGGAGGGCCTTACTAATTACTTA; hprt-intron2-R2: ATATGTACTTTTGCATATAGTATAC; olMR0015: AAATGTTGCTTGTCTGGTG; and olMR0016: GTCAGTCGAGTGCACAGTTT. For *Rbp4-Cre* mice: Rbp4 (31125) F: GGGCGGCCTCGGTCCTC, GS Cre R2: CCCCAGAAATGCCAGATTACGTAT. The PCR conditions were as follows: pre-denaturation at 95 °C for 2min, followed by 30 amplification cycles of denaturation at 95 °C for 30s, primer annealing at 58 °C for 30s, extension at 72 °C for 3s, and a final extension at 72 °C for 5min. Littermate and age-matched wild-type (WT) mice were used as controls. A total of 214 mice were used in this study, including 89 WT, 88 *15q dup*, 16 *Rbp4-Cre*, and 21 *15q dup; Rbp4-Cre* mice. All animals were age-matched littermates and were utilized across the experiments described below. All the mice were maintained under specific pathogen-free conditions in the transgenic mouse room of the Department of Experimental Animals, Interdisciplinary Center for Science Research, Head Office for Research and Academic Information, Shimane University, Japan.

### Immunofluorescence study

Immunostaining was performed as previously described [28, 29]. The brains were collected from 8-week-old mice anesthetized with an intraperitoneal injection of a mixture containing 0.3mg/kg medetomidine, 4.0mg/kg midazolam, and 5.0mg/kg butorphanol. Cardiac perfusion fixation was performed using either 4% paraformaldehyde (PFA) in 0.01M phosphate-buffered saline (PBS; pH 7.4) for Ankyrin-G or 3% glyoxal/0.8% acetic acid in 0.1M phosphate buffer (pH 4.0) for pan-voltage-gated sodium (Pan-Nav) channels. After perfusion, post-fixation was performed for 2 (PFA) or 24h (glyoxal). Then, brains were rinsed with saline and cryoprotected overnight in 30% sucrose in PBS at 4 °C. Coronal brain sections (40 μm thickness) were cut using a sliding microtome (HM430; Thermo Fisher Scientific, Waltham, MA, USA).

Brain slices were pre-incubated for 1h in 0.01M PBS (pH 7.4) containing 0.4% Triton X-100 and 3% normal donkey serum (PBTDS). Subsequently, the slices were incubated for two nights at 4 °C with primary antibodies diluted in PBTDS. After thorough rinsing with PBST (PBS containing 0.1% Triton X-100), the samples were incubated overnight at 4 °C with the appropriate fluorescent secondary antibodies. Finally, the samples were washed every 5min with PBST, mounted onto gelatin-coated glass slides and coverslips (Matsunami Glass, Osaka, Japan) using VECTASHIELD PLUS Antifade Mounting Medium (Vector Laboratories, Burlingame, CA, USA), and examined under FV-1000D and FV-3000D confocal microscopes (Olympus, Tokyo, Japan). The antibodies and reagents used are listed in the supplementary materials.

### AIS imaging and analysis

AIS imaging and analyses were performed as previously described [29]. All AIS images were obtained using immunofluorescence. To minimize nonspecific background signal, antibody concentrations and incubation conditions were optimized, and images were acquired using low laser power and detector gain settings. Background subtraction was applied uniformly across all images, and a linear look-up table was used to enhance signal-to-noise contrast. All images were acquired using FV-1000D and FV-3000D confocal microscopes (Olympus, Tokyo, Japan) with a 60x oil-immersion objective lens (Numerical Aperture [NA] = 1.30). To quantify the AIS structure, four fields of view were captured for each brain region. Each confocal image was acquired at a resolution of 1024 × 1024 pixels, with an XY resolution of 4.9010 pixels/μm and a Z-stack interval of 1 μm. All AIS lengths in the 3D image stacks were automatically measured using the Simple Neurite Tracer plugin in ImageJ, after manually specifying the start and end points of each AIS in the Z-stack images [30].

### Puncta quantification

Puncta quantification was carried out in layers II/III and V within several cortical regions [23], including the prelimbic (PrL), infralimbic (IL), somatosensory (SC), and motor (MC) cortices. Imaging of 8-week-old male mice was performed using an Olympus FV3000D confocal microscope equipped with a 60× objective and an additional 1.6× digital zoom, with a Z-stack interval of 1 μm. For Synaptophysin-1-positive presynaptic puncta measurements, 15 images (512 × 512 pixels) were obtained from five mice per genotype. Regions of interest within the measurement area that were devoid of neuronal somata were manually delineated using ImageJ. These regions were thresholded to generate binary representations, with threshold parameters adjusted to ensure consistent detection of punctate signals across samples. To verify that the thresholding procedure did not introduce bias, we examined whether puncta count increased proportionally with the number of optical sections collected at 1 μm spacing. All imaging and quantification were performed under blinded conditions with respect to genotype.

### Electrophysiology

Brains were rapidly removed and immersed in ice-cold artificial cerebrospinal fluid (ACSF) containing 124mM NaCl, 2.5mM KCl, 2mM CaCl_2_, 1mM MgSO_4_, 25mM NaHCO_3_, 1mM NaH_2_PO_4_, and 10mM glucose bubbled with carbogen gas (95% O_2_, 5% CO_2_). Coronal brain slices (300µm thickness), including the medial prefrontal cortex (mPFC) region, were prepared using a vibratome (7000smz-2; Campden, Loughborough, Leicestershire, England) [31]. Slices were incubated in ACSF saturated at 25 °C for approximately 1h. Recordings were conducted in a chamber mounted on a BX51WI microscope (Olympus, Center Valley, PA, USA) using infrared differential interference contrast optics for neuron visualization. Whole-cell patch-clamp recordings were performed on layer V pyramidal neurons (PyNs) in the PrL and IL cortices using an amplifier (Axopatch 200B; Molecular Devices, San Jose, CA, USA) at 25 °C. The recording pipettes were pulled from borosilicate glass (outer diameter: 1.5mm; inner diameter: 1.12mm; World Precision Instruments, Sarasota, FL, USA) to achieve a tip resistance of 2–5 MΩ. The pipette solution contained 120 mM K-gluconate, 5mM NaCl, 1mM MgCl₂, 0.5mM EGTA, 2mM Mg-ATP, 0.1mM Na₃GTP, and 10mM HEPES (pH 7.2, 280–300mOsm), which was used to measure resting membrane potentials (RMPs), action potentials (APs), and spontaneous excitatory postsynaptic currents (sEPSCs). The RMPs were recorded immediately after obtaining the whole-cell configuration in current-clamp mode (I=0). AP properties were assessed using 20-ms step pulses in 10-pA increments from a holding current of I=0. For the firing pattern and input-output (I–F) curve analysis, 500ms current steps increasing by 50 pA were applied to elicit AP trains. The slope and peak of each I–F curve were calculated for each group. sEPSCs were recorded for 3min in the voltage-clamp mode while holding the membrane potential at -70 mV. APs and sEPSCs were analyzed using Mini Analysis Software, and membrane potential properties were analyzed using Clampfit 10.7 (Molecular Devices). A summary of the electrophysiological parameters is presented in Supplementary Tables.

### AIS plasticity analysis

Acute brain slices were prepared as previously described [29], with minor changes. The coronal slices (300 μm thick) were incubated in a storage chamber filled with carbogen-saturated ACSF at 37 °C, with or without 8mM KCl for depolarization, for either 1 or 3h. After depolarization, the slices were post-fixed in 4% PFA for 30min on ice. The tissue was then rinsed with saline and cryoprotected overnight in 30% sucrose in phosphate-buffered saline (PBS) at 4 °C. Brain sections (40 μm thick) were cut using a cryostat (CM1900; Leica, Wetzlar, Germany), mounted on FRONTIER glass slides (Matsunami Glass, Osaka, Japan), and stored at -20 °C until use. The slices were pre-incubated for 1h in PBTDS. The samples were then incubated with primary antibodies diluted in 0.01M PBS (pH 7.4) containing 0.4% Triton X-100 and 3% normal goat serum (PBTGS) for 40h at 4 °C. After thorough rinsing with PBST, fluorescently labeled secondary antibodies were applied overnight at 4 °C. Finally, the samples were washed every 5min with 0.01M PBST, mounted with VECTASHIELD PLUS Antifade Mounting Medium (Vector Laboratories), and imaged using FV-1000D and FV-3000D confocal microscopes (Olympus).

### Western blotting

mPFC homogenates were prepared from *15q dup* and WT mice. All procedures were performed on ice or at 4 °C. The mPFC was homogenized using an ultrasonic homogenizer (SONIFIER 450 Advanced; Branson Ultrasonics Corporation, Danbury, CT, USA) in radioimmunoprecipitation assay (RIPA) buffer (Wako, Osaka, Japan) supplemented with 2M EGTA and a Protease Inhibitor Cocktail Set III (Wako, Osaka, Japan). The homogenates were centrifuged at 800×*g* for 10min to remove chromosomal DNA, cellular debris, and fibers. The resulting supernatants were collected and stored at –80 °C as whole homogenate fractions. Protein concentrations were determined using the bicinchoninic acid (BCA) assay (Thermo Fisher Scientific). Samples were subjected to SDS–PAGE and immunoblotting using standard protocols. Western blotting was performed as previously described [28] with minor modifications. Proteins were separated on 5–20% gradient e-PAGEL mini gels (ATTO Corporation, Tokyo, Japan) and transferred onto Immobilon-P polyvinylidene fluoride (PVDF) membranes (Merck Millipore, Billerica, MA, USA). Membranes were blocked for 30min with Blocking One (Nacalai Tesque, Kyoto, Japan) and incubated overnight with primary antibodies diluted in 5% Blocking One in Tris-buffered saline containing 0.1% Tween-20 (T-TBS). After three washes with T-TBS, the membranes were incubated with secondary antibodies at 25 °C followed by three additional washes. Signals were detected using an enhanced chemiluminescence (ECL) system (Cytiva, Tokyo, Japan). Chemiluminescent signals and pre-stained molecular weight markers were imaged simultaneously using an ImageQuant 800 system (Amersham/Cytiva, Tokyo, Japan). Band intensities were quantified using the ImageJ software (https://imagej.net/welcome). Full blot images are shown in the Supplementary information. All the antibodies used are listed in the supplementary materials.

### RNA extraction and quantitative real-time PCR

RNA extraction and quantitative real-time PCR were performed as previously described [28] with slight modifications. The mouse brains were dissected and immediately snap-frozen in liquid nitrogen. Frozen tissue was powdered using BioMasher II (Nippi, Tokyo, Japan) and total RNA was extracted using Isogen II reagent (Nippon Gene, Tokyo, Japan).

First-strand cDNA was synthesized from total RNA using ReverTra Ace qPCR RT Master Mix with gDNA Remover (Toyobo, Osaka, Japan). mRNA expression was quantified using THUNDERBIRD NEXT SYBR qPCR Mix (Toyobo, Osaka, Japan) with a Thermal Cycler Dice Real-Time System II TP900 (Takara Bio, Shiga, Japan). The relative mRNA levels of target genes were normalized to those of β-actin. The copy numbers were calculated using standard calibration curves for each gene of interest. The primer sequences used are listed in the supplementary materials.

### Retrograde tracing

Retrograde tracing was performed via stereotaxic injection of cholera toxin B subunit (CTB; List Biological Labs, Campbell, CA, USA) into the nucleus accumbens (NAcc: A=+1.30, L=±0.85, D = −3.80), ventromedial caudate putamen (CPVM: A=+0.26, L=±1.25, D = −3.00), lateral habenula (LHb: A = −1.40, L=±0.75, D = −3.90), ventral tegmental area (VTA: A = −3.30, L=±0.40, D = −4.00), and dorsal raphe nucleus (DRN: A = −4.24, L = −1.10, D = −2.85 at a 20° angle) in WT and *15q dup* mice. 50 nL of 0.5% CTB diluted in saline was injected using glass micropipettes. Five days after the CTB injection, the mice were anesthetized by intraperitoneal injection of a mixture containing 0.3mg/kg medetomidine, 4.0mg/kg midazolam, and 5.0mg/kg butorphanol. Cardiac perfusion fixation was performed using 4% PFA in 0.01M PBS. Post-fixed brains were rinsed with saline and cryoprotected overnight in 30% sucrose in PBS at 4 °C. Coronal brain sections (40 μm thick) were prepared using a sliding microtome (HM430; Thermo Fisher Scientific, Waltham, MA, USA). Immunostaining was performed as described previously [29].

### Adeno-associated virus (AAV) production

For chemogenetic experiments, we utilized designer receptors exclusively activated by designer drugs (DREADD) [32]. The pAAV-nEF Con/Fon DREADD Gq-mCherry vector, deposited by Karl Deisseroth, was obtained from Addgene (RRID: Addgene_183532), and the pUCmini-iCAP-AAV9-X1.1 plasmid, deposited by Viviana Gradinaru, was obtained from Addgene (RRID: Addgene_196836). Recombinant single-stranded AAV9 vectors were generated using a modified ultracentrifugation protocol [33]. HEK293T cells (RRID: RCB2202) were cultured in 10cm dishes in Dulbecco’s modified Eagle’s medium (DMEM; Wako, Osaka, Japan) supplemented with 10% fetal bovine serum (Hyclone/Cytiva, Tokyo, Japan). Cells were transfected with three plasmids, pAAV-nEF Con/Fon DREADD Gq-mCherry, pUCmini-iCAP-AAV9-X1.1, and pHelper (Agilent Technologies, Santa Clara, CA, USA), using polyethyleneimine Max (Polysciences, Inc., Warrington, PA, USA) as the transfection reagent. The culture medium was replaced with serum-free DMEM to enhance viral production 24h after transfection. Five days post-transfection, the culture medium containing AAV particles was collected and centrifuged at 800×*g* for 5min to remove cell debris. AAV particles were precipitated using 8% polyethylene glycol (PEG) 8000 (Sigma-Aldrich, St. Louis, MO, USA) with 0.5M sodium chloride for 2h at 4 °C, and then centrifuged at 3200×*g* for 60min at 4 °C. The pellet was resuspended in PBS containing 2.6mM MgCl₂ and treated with 26U/mL of benzonase (Merck Millipore, Darmstadt, Germany) at 37 °C for 30min. The benzonase-treated solution was ultracentrifuged through a discontinuous iodixanol gradient (15%, 25%, 40%, and 60% OptiPrep; Serumwerk Bernburg AG, Bernburg, Germany) at 60,000×*g* for 90min at 10 °C using an L-60 ultracentrifuge and a 60Ti rotor (Beckman Coulter, Brea, CA, USA). A 5mL fraction from the 40% iodixanol layer was collected and diluted with 1mL of PBS. To concentrate the viral particles, the diluted solution was transferred to a Vivaspin 20 column (Sartorius, Göttingen, Germany) pre-blocked with 1% bovine serum albumin (BSA) in PBS. The column was centrifuged at 2600×*g* for 60min at 4 °C. The viral solution was washed twice with 15mL of PBS, and the final volume was adjusted to approximately 200μL. Viral titers were determined using quantitative PCR.

### Stereotaxic injection of AAV

The AAV-retrograde-EF1a-Flpo vector (hereafter referred to as AAV-retro-Flpo), originally developed by Karl Deisseroth’s laboratory, was obtained from Addgene (RRID: Addgene_55637). Stereotaxic injections of AAV-retro-Flpo combined with 0.1% CTB into the DRN (A = −4.24, L = −1.10, D = –2.85 at a 20° angle) and AAV9-nEF Con/Fon DREADD Gq-mCherry into the mPFC (A=+1.98, L=±0.30, D = –1.30) were administered to WT, *15q dup*, *Rbp4-Cre*, and *15q dup*; *Rbp4-Cre* mice at 8 weeks of age. 100 nL of AAV solution diluted in saline was injected using glass micropipettes. Mice were anesthetized via intraperitoneal injection of a combination of 0.3mg/kg medetomidine, 4.0mg/kg midazolam, and 5.0mg/kg butorphanol. Behavioral testing was conducted four weeks after AAV injection. Following behavioral assessment, the mice were transcardially perfused with 4% PFA in 0.01M PBS. Brains were post-fixed, rinsed in saline, and cryoprotected in 30% sucrose in PBS at 4 °C overnight. Coronal brain sections (40 μm thick) were cut using a sliding microtome (HM430; Thermo Fisher Scientific, Waltham, MA, USA), and immunostaining was performed as described previously [29].

### Three-chamber test (3-CT)

The 3-CT was based on the three-phase protocol described by Rein et al.[34] with minor modifications to the original protocol. The protocol consisted of the following phases: habituation, pre-test, social preference, and social novelty. All the mice were habituated to the behavioral testing room for 1h prior to testing. To activate the DREADD system, deschloroclozapine (DCZ) was administered intraperitoneally (0.03mg/kg) 1h before the test [35]. The 3-CT apparatus measured 42 × 60 × 25cm and contained two transparent cups (SHINFACTORY, Fukuoka, Japan) for stimulus placement. During the habituation phase, the mice were placed in the center chamber with open doors and were allowed to explore the empty apparatus for 10min. In the pre-test phase, two clean paper balls were placed in each cup to habituate the mice to the presence of the objects. The mice were allowed to explore the area for 10min. In the social preference phase, a novel age-, sex-, and strain-matched WT mouse (social stimulus (S1)) was placed in one cup, and a wooden block (2.5cm square; non-social stimulus (NS)) was placed in the other. The mice were allowed to explore the area for 10min.

In the social novelty phase, the wooden block was replaced with a second unfamiliar WT mouse (social stimulus S2) while the first mouse (S1) remained in the original cup. The test mice were allowed to explore the area for 10min. The sniffing time for each stimulus was recorded using ANY-maze v7.34 (Stoelting Co., Wood Dale, IL, USA), and social preference and novelty indices were calculated based on the interaction times.

### Marble-Burying Test (MBT)

MBT was conducted according to the method described by Njung’e et al. [36], with minor modifications. All the mice were habituated to the behavioral testing room for 1h prior to testing. DCZ was administered intraperitoneally (0.03mg/kg) 1h before the test to activate the DREADD system. Each mouse was individually placed in a test cage (44 × 27 × 20cm) filled with 5cm of wooden bedding and allowed to explore freely for 30min for acclimatization. After habituation, the mice were temporarily returned to their home cages for a 5min rest period. During this period, 20 clean glass marbles (1.5cm diameter) were evenly placed on the surface of the bedding in the test cage. The mice were then gently reintroduced into the test cage and allowed to explore the cage for 30min. After each session, the number of marbles buried at least two-thirds of the bedding depth was recorded. All tests were recorded as videos for the analysis.

### Statistical analyses

All statistical analyses were performed using Prism 9 (GraphPad Software, La Jolla, CA, USA). Data are expressed as mean±standard error of the mean (SEM). Comparisons between the two groups were conducted using Student’s t-test or the Mann–Whitney U test. For comparisons between more than two groups, one-way analysis of variance (ANOVA) followed by Tukey’s post-hoc test, repeated-measures two-way ANOVA, or two-way ANOVA followed by Tukey’s or Sidak’s multiple comparison tests were used, as appropriate. Effect sizes (eta squared, η²) for ANOVA were calculated using the following formula: η^2^=SSA/SST, where SSA is the sum of squares between groups and SST is the total sum of squares. Statistical significance was set at *P*<0.05. All experiments were performed under blinded conditions. Detailed statistical information, including test statistics (t or F values), degrees of freedom, exact P values, and the results of main effects and interactions, is reported in the corresponding supplementary Tables to ensure transparency and reproducibility.

## Results

### Altered AIS structure and lack of plastic responsiveness to KCl-induced depolarization in cortical PyNs of *15q dup* mice

**Fig. 1:**
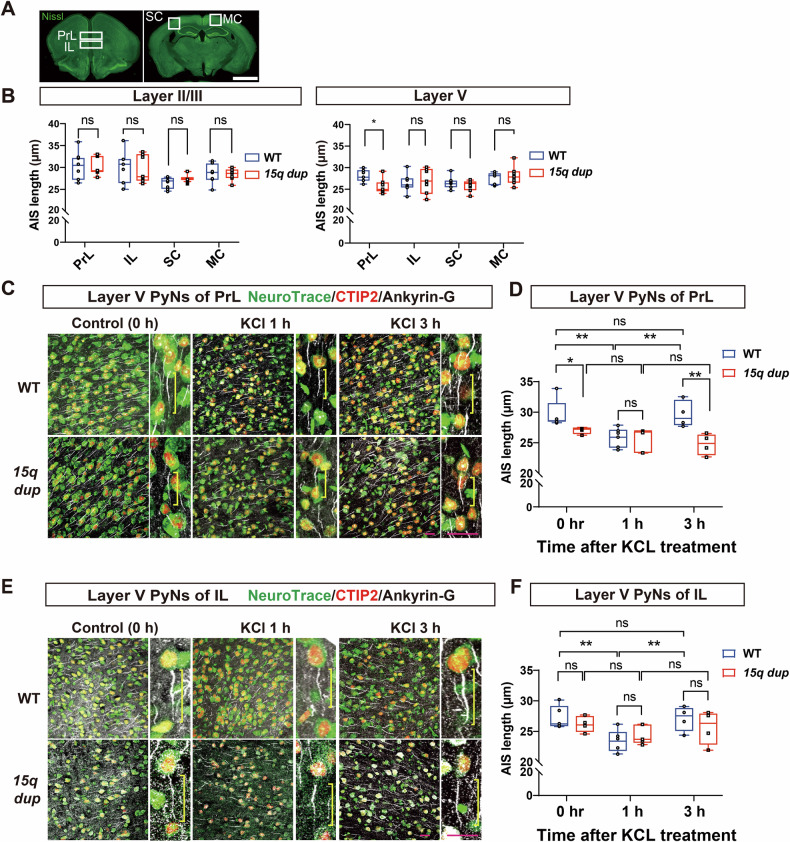
Altered axon initial segment (AIS) structure and a lack of plastic responsiveness to KCl-induced depolarization in layer V pyramidal neurons of *15q dup* mice. **A** Representative coronal brain sections showing the analyzed cortical regions: prelimbic cortex (PrL), infralimbic cortex (IL), somatosensory cortex (SC), and motor cortex (MC). **B** Quantification of AIS length in layer II/III and layer V pyramidal neurons (PyNs). Values represent medians with 25–75% intervals, and the error bars indicate the minimum-to-maximum values with individual data points (*n* = 7 mice per group; >50 AIS per mouse). **C**, **E** Representative immunofluorescence images showing AIS plasticity in PrL (C) and IL (E) following KCl treatment in wild-type (WT) and *15q dup* mice. **D**, **F** Quantification of AIS length changes over time (*n* = 4–6 slices per group; >50 AIS per mouse). Values represent medians with 25–75% intervals, and the error bars indicate the minimum-to-maximum values with individual data points. Scale bars: 100 μm (**A**), 10 μm (**C**, **E**). A summary of all statistical values is provided in Table S1.

We recently developed a precise method to measure AIS length in vivo (Fig. S1) [29]. We visualized PyNs by immunohistochemically detecting Ankyrin-G as an AIS marker, in combination with layer-specific markers and a neural cell body marker (fluorescent Nissl). We selected the following cortical focus areas in *15q dup* mice: the PrL and IL regions of the mPFC, as well as the SC and MC (*n*=7 mice per group; >50 AIS per mouse in layers II/III and V; Figs. 1A, B and S1). We quantified AIS length in layers II/III and V PyNs of WT and *15q dup* mice (Figs. 1B and S1). We observed that AIS length varied by cell layer and cortical location. In layer II/III PyNs, AIS length was unchanged across all examined areas, with no significant genotype effect observed. In contrast, layer V PyNs showed a significant decrease in AIS length specifically in the PrL, with no significant differences observed in the IL, SC, or MC (Figs. 1B and S1A, B). We analyzed the cumulative frequency distributions to confirm this reduction at the population level. Notably, *15q dup* mice only exhibited a significant leftward shift relative to WT controls in layer V of the PrL, indicating a robust reduction in AIS length (Fig. S1C). These findings revealed a significant reduction in AIS length, specifically in layer V PyNs of the PrL in *15q dup* mice, suggesting that AIS alterations in this ASD model were region- and layer-specific. All statistical details for these comparisons are available in Tables S1 and S2.

We next assessed activity-dependent AIS plasticity in vitro using acute mPFC slices and high-concentration KCl depolarization [9]. In WT mice, layer V PyNs in the PrL and IL exhibited robust plasticity: KCl exposure induced significant AIS shortening at 1h, which returned to baseline length by 3h (each time point *n*=4–6 slices per group; >50 AIS per mouse in PrL and IL; Fig. 1C–F). We then tested whether this plasticity was maintained in *15q dup* mice. In the PrL, AIS length was significantly shorter in *15q dup* mice at baseline (0h) than in WT controls. Notably, these neurons showed no further shortening after 1h of KCl exposure and failed to recover at 3h, demonstrating a clear lack of plasticity (Fig. 1D). A similar impairment was observed in IL PyNs of *15q dup* mice (Fig. 1F).

These results indicate that, whereas in vivo shortening was restricted to the PrL, in vitro impairment of AIS plasticity occurred broadly across layer V PyNs of the mPFC in *15q dup* mice. All statistical details for these comparisons, including two-way ANOVA and post-hoc analyses, are available in Table S1.

### Altered electrophysiological properties in layer V PyNs in the PrL of *15q dup* mice, but not in the infralimbic cortex (IL)

**Fig. 2:**
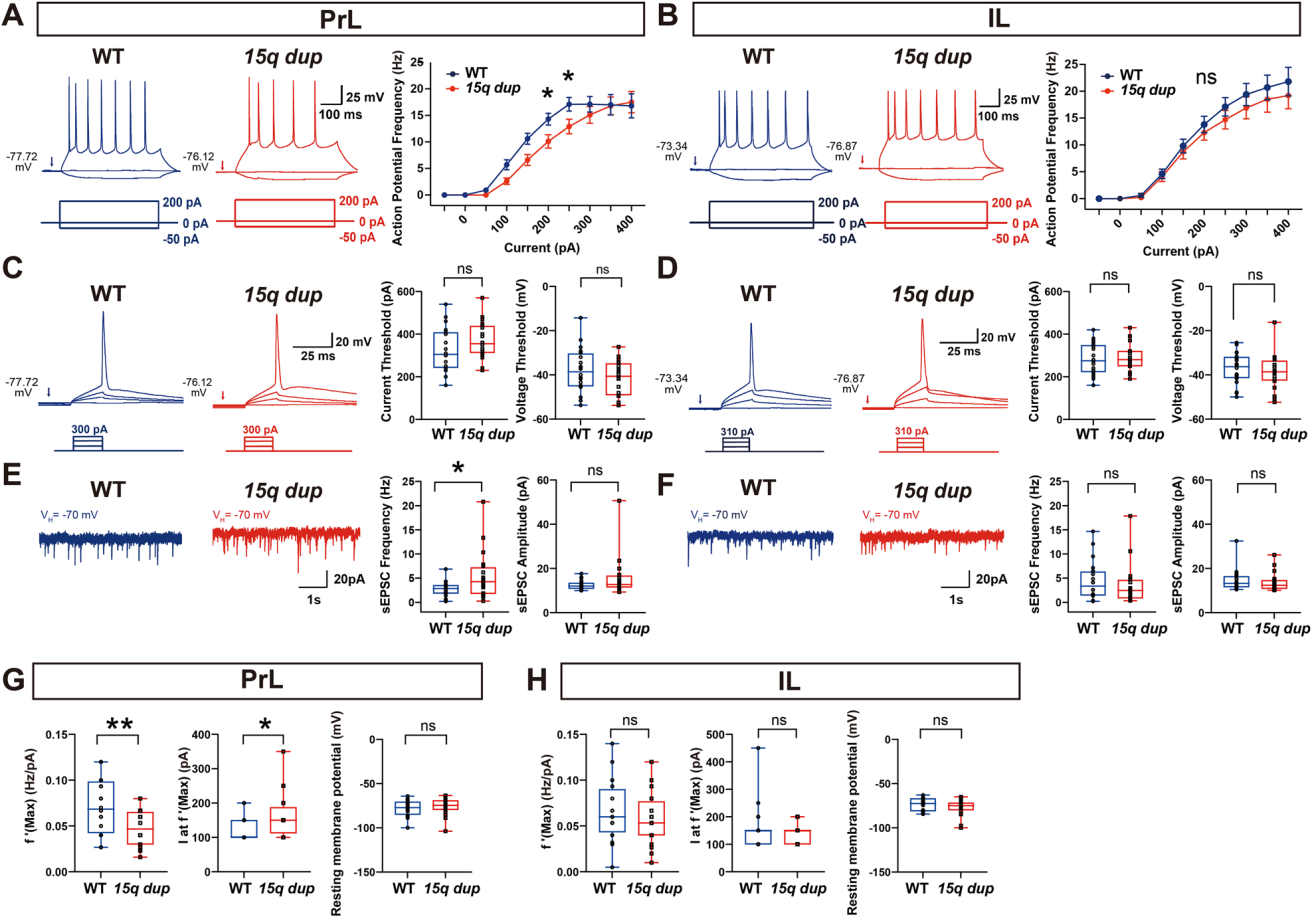
Specific alteration of electrophysiological properties in layer V pyramidal neurons (PyNs) in the prelimbic cortex (PrL) of *15q dup* mice. **A**, **C**, **E**, and **G** (left panels) show the analysis of PrL PyNs, and **B**, **D**, **F**, and **H** (right panels) show the analysis of the infralimbic cortex (IL). **A**, **B** Representative traces of action potential (AP) trains evoked by current injection (500ms, −50 pA, 0 pA, and +400 pA). The AP frequency was determined using 500ms injections of the indicated increasing current. All values are presented as means±standard error of the mean (SEM) and were analyzed using repeated-measures two-way analysis of variance (ANOVA) with Sidak’s multiple comparisons test. ns, not significant, *P<0.05. Data represent *n*=20 cells obtained from four biologically independent experiments per group. **C**, **D** Representative traces of single APs. The current and voltage thresholds were measured using a step protocol of 20-ms pulses in 10-pA increments. All values are presented as medians with 25–75% intervals, and the error bars indicate the minimum-to-maximum values with individual data points. ns, not significant. Statistical analysis was performed using a two-tailed unpaired Student’s *t* test (*n*=20 cells from four biologically independent experiments). **E**, **F** Representative traces of spontaneous excitatory postsynaptic currents (sEPSCs). sEPSCs were recorded with a holding membrane potential at −70mV in the voltage-clamp mode for 3min. All values are presented as medians with 25–75% intervals, and the error bars indicate the minimum-to-maximum values with individual data points. ns, not significant; *P<0.05, analyzed using a two-tailed unpaired Student’s *t* test (*n*=20 cells from four biologically independent experiments). **G**, **H** Analysis of the maximum slope fʹ(max) of the I-f curve, the current at the maximum slope I at fʹ(max), and the resting membrane potential. All values are presented as medians with 25–75% intervals, and the error bars indicate the minimum and maximum values with individual data points. ns, not significant; **P<0.01, *P<0.05, analyzed using a two-tailed unpaired Student’s *t* test (*n*=20 cells from four biologically independent experiments). A summary of all statistical values is provided in Table S3.

Next, we performed whole-cell patch-clamp recordings on acute mPFC slices to determine whether the structural AIS shortening corresponded to decreased neuronal excitability. Indeed, we found that AP frequency in response to depolarizing current steps was significantly decreased in layer V PyNs in the PrL of *15q dup* mice but not the IL (*n*=20 cells from 4 biologically independent mice; Fig. 2A, B). Consistent with this, fʹ(Max), the maximum slope of the I–f curve, was also significantly decreased in *15q dup* mice, specifically in the PrL (Fig. 2G). Intriguingly, these hypoexcitable PrL neurons exhibited a concurrent increase in the frequency of sEPSCs (Fig. 2E). Other passive properties were unaltered, including RMP, current and voltage thresholds, and sEPSC amplitude (Fig. 2C, E, G). Notably, no reduction in AP firing or increase in sEPSC frequency was observed in the adjacent IL (Fig. 2B, D, F, H). These results reveal a paradoxical physiological state specific to PrL layer V PyNs in the *15q dup* model: they receive stronger excitatory synaptic inputs yet exhibit a reduced capacity for AP generation, a homeostatic adaptation likely mediated by AIS shortening. All statistical details for these electrophysiological properties are available in Table S3.

To determine whether the observed increase in sEPSC frequency was driven by structural changes in presynaptic inputs, we quantified Synaptophysin-1-positive presynaptic boutons across multiple cortical regions, including PrL, IL, SC and MC, in both layers II/III and V. Across all examined regions and layers, the density of Synaptophysin-1-positive presynaptic puncta did not differ between genotypes (*n*=5 mice per group; Fig. S2A, B), indicating that presynaptic bouton number and thus local network connectivity at the presynaptic level remains preserved in the *15q dup* mice. All statistical details for these comparisons are available in Table S4.

### AIS shortening occurs independently of changes in the expression of core AIS components

Next, we examined whether AIS length alterations were associated with a misdistribution between voltage-gated sodium (Nav) channels and Ankyrin-G. Pan-Nav channels colocalized with Ankyrin-G (Fig. S3A, B). No significant difference in length between clusters of Pan-Nav and Ankyrin-G was found in the affected PrL layer V or in other regions (*n*=4 mice per group; >50 AIS per mouse in layers II/III and V; Figs. S3A, B and S4; S5).

Similarly, we quantified Ankyrin-G and Pan-Nav protein levels in the PrL using western blot analysis and found that the expression levels of the main AIS-related Ankyrin-G isoforms (270 and 480kDa) and Pan-Nav channels were unchanged in *15q dup* mice (*n*=6 mice per group; Figs. S3C and S6). However, we found a significant decrease in the 190kDa Ankyrin-G isoform, a dendrite-localizing isoform (Fig. S3C) [37]. Next, we examined transcript levels of AIS components using real-time polymerase chain reaction (PCR). No significant changes were observed in *Ankyrin-G, β4-spectrin, Nav1.2, or Nav1.6* transcripts (*n*=6 mice per group; Fig. S3D), indicating that AIS length changes in *15q dup* mice were not attributable to changes in Ankyrin-G or Nav channels at the protein and transcript levels.

Furthermore, we investigated whether AIS length changes could result from abnormal microtubule stability, as observed in FTD patients [17]. No significant EB3 accumulation was observed in the AIS of either group (Fig. S3E), suggesting that AIS modifications are dependent on neuronal activity rather than microtubule stability. All statistical details for these biochemical and structural comparisons are available in Tables S5, S6, S7.

### AIS length of PyNs projecting to specific areas is significantly decreased in *15q dup* mice

**Fig. 3:**
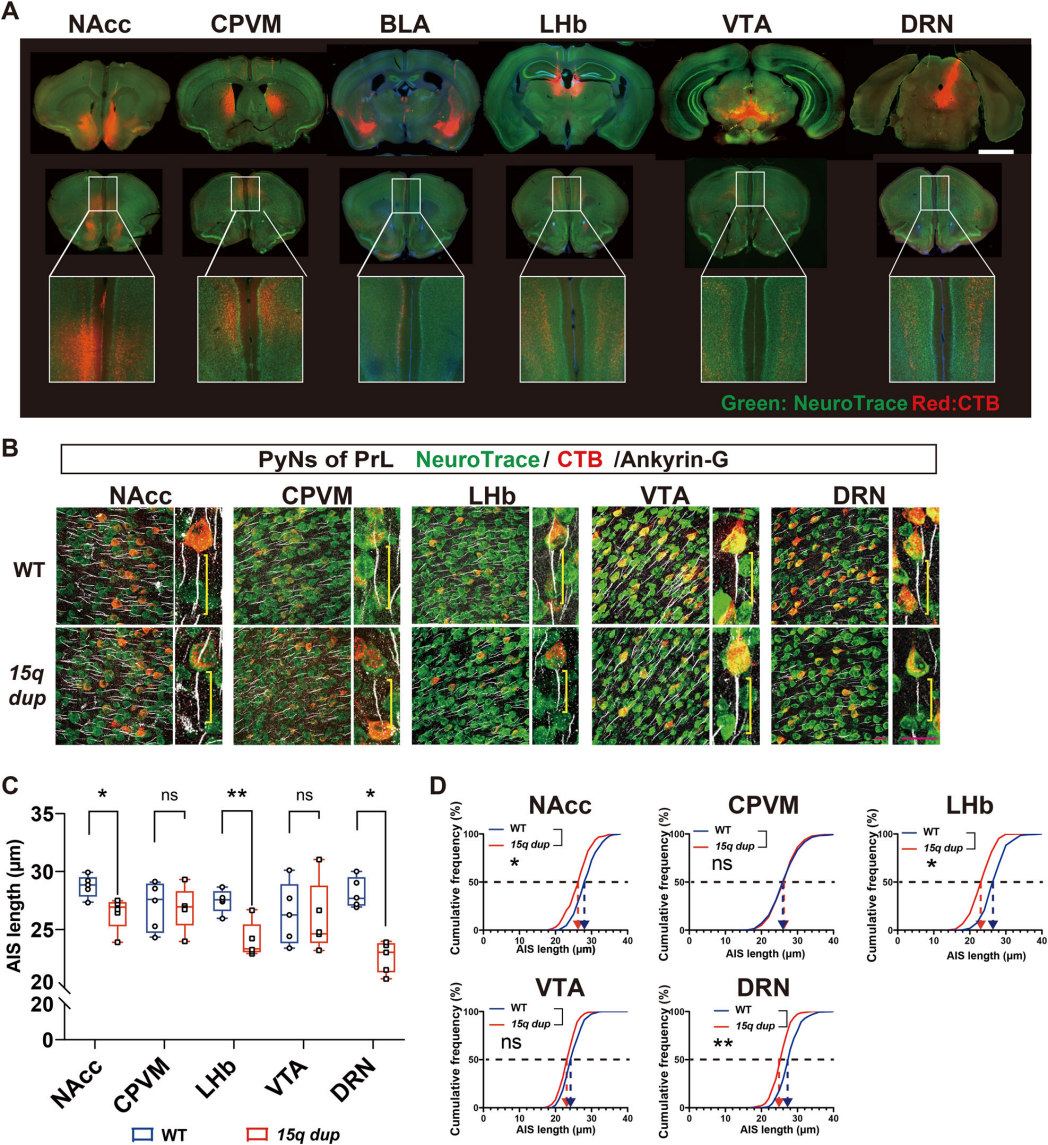
The length of the axon initial segment (AIS) of layer V pyramidal neurons (PyNs) in the prelimbic cortex (PrL) of *15q dup* mice varies depending on the projection target. **A** Upper: Representative retrograde tracing images of NeuroTrace® (green) and cholera toxin B subunit (CTB) (red). CTB was injected into the nucleus accumbens (NAcc), ventromedial caudate putamen (CPVM), lateral habenula (LHb), ventral tegmental area (VTA), and dorsal raphe nucleus (DRN). Lower: Representative image of the projected area corresponding to the medial prefrontal cortex (mPFC). Scale bar = 100µm. **B** Representative confocal images of the AIS in layer V PyNs of wild-type (WT) and *15q11–13 duplication* (*15q dup*) mice. Fluorescent colors indicate NeuroTrace®(green), CTB (red), and Ankyrin-G (white). Scale bar = 10µm. **C** Analysis of AIS in layer V PyNs in the mPFC projecting to the NAcc, CPVM, LHb, VTA, and DRN in WT and *15q dup* mouse brains. All values are presented as medians with 25–75% intervals, and the error bars indicate the minimum-to-maximum values with individual data points. *P<0.05, **P<0.01, analyzed using two-way analysis of variance (ANOVA) with Sidak’s multiple comparisons tests (*n*=5 mice per group; >50 AIS per mouse). **D** Analysis of the cumulative frequency of AIS length in layer V PyNs in the mPFC projecting to the NAcc, CPVM, LHb, VTA, and DRN in WT (blue) and *15q dup* (red) mouse brains. The arrows indicate the AIS length at 50% of the cumulative frequency distribution curve. Statistical analyses were performed using the Mann–Whitney U test (*n*=5 mice). ns, not significant; *P<0.05, **P<0.01. A summary of all statistical values is provided in Table S8.

Next, we investigated whether AIS shortening in PrL layer V PyNs was uniform or specific to defined long-distance circuits. We used retrograde tracing with CTB to label neurons by their projection targets. Based on previous reports [38, 39], we stereotaxically injected CTB into five major layer V projection areas: NAcc, CPVM, LHb, VTA, and DRN (*n*=5 mice per group; >50 AIS per mouse in each area; Fig. 3A, B). We noted that projections to the basolateral amygdala (BLA) originated predominantly from layer II/III PyNs, and therefore focused the layer V analysis on the other five targets.

Analysis of retrogradely labeled neurons revealed a striking, target-specific vulnerability (Fig. 3C). AIS length was significantly decreased in *15q dup* PyNs projecting to the NAcc, LHb, and DRN, but remained unchanged in neighboring neurons projecting to the CPVM and VTA compared with WT controls. Cumulative frequency distribution analysis confirmed this projection-specific reduction (Fig. 3D). A significant leftward shift in AIS length distribution was observed only in the affected NAcc-, LHb-, and DRN-projecting populations, while CPVM- and VTA-projecting populations were indistinguishable from controls.

These results indicate that abnormal AIS length in *15q dup* mice is not a general cellular defect but reflects target-specific neural circuit abnormalities. All statistical details for these comparisons, including two-way ANOVA and post-hoc analyses, are available in Table S8.

### Circuit-specific chemogenetic activation rescues AIS shortening

Given that AIS shortening was specific to layer V PyNs projecting to the NAcc, LHb, and DRN, we next investigated whether this structural pathology was reversible. We focused on the mPFC–DRN pathway, as abnormal 5-HT production in the DRN has been directly linked to ASD-like behaviors in *15q dup* mice [23].

**Fig. 4:**
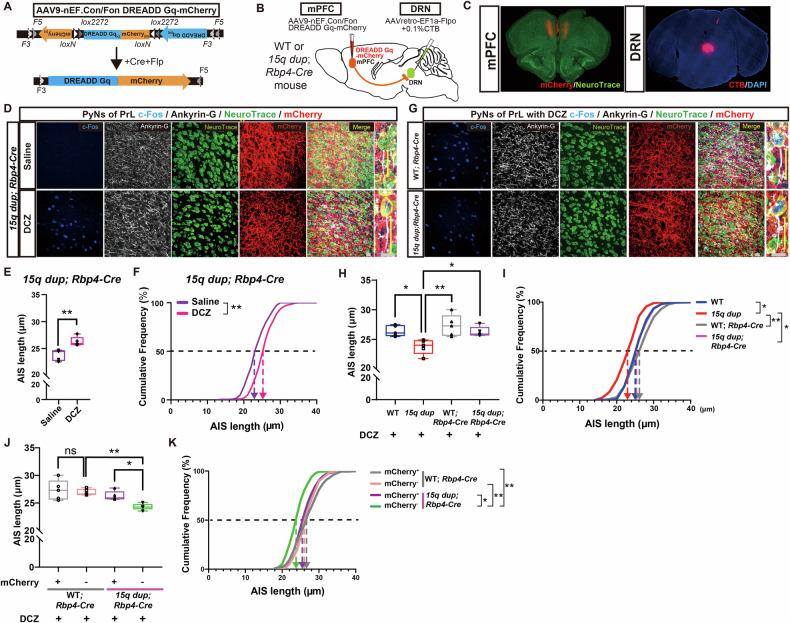
Recovery of axon initial segment (AIS) length in pyramidal neurons (PyNs) projecting to the dorsal raphe nucleus (DRN) in the medial prefrontal cortex (mPFC) by the activation of designer receptors exclusively activated by designer drugs (DREADD) in *15q dup*; *Rbp4-Cre* mice. **A** Diagram of the Con/Fon DREADD Gq-mCherry sequence split into three exons with *loxN, lox2272, FRT3 (F3)*, and *FRT5 (F5)* recombinase recognition sites (top). The activity of Cre and Flpo recombinases repositions the exons in the correct sense direction, resulting in an intact mRNA encoding DREADD Gq-mCherry. **B** Schematic image of the Con/Fon target-specific neurocircuit expression system and stereotaxic injection strategy of adeno-associated virus (AAV) vector encoding Con/Fon DREADD Gq-mCherry injected into the mPFC and AAV retro-Flpo + 0.1% cholera toxin B subunit (CTB) injected into the DRN. **C** Representative fluorescence image of successful AAV injection. AAV9 Con/Fon DREADD Gq-mCherry (top) showed expression in the mPFC when injected with AAV retro-Flpo + 0.1% CTB into the DRN (bottom). **D** Representative confocal images of c-Fos (blue), Ankyrin-G (white), NeuroTrace® (green), and mCherry (red) in layer V PyNs of the prelimbic cortex (PrL) in *15q dup*; *Rbp4-Cre* mice with saline or 0.03mg/kg deschloroclozapine (DCZ) administered 60min before sacrifice. Gray bars indicate AIS. Scale bar = 10µm. **E**, **F** Quantification of AIS length (**E**: *n*=5 mice per group; >50 AIS per mouse) and cumulative frequency of AIS length (**F:** *n*=5 mice per group**)** in layer V PyNs with saline or DCZ treatment in *15q dup*; *Rbp4-Cre* mice. Values are presented as medians with 25–75% intervals, and the error bars indicate the minimum-to-maximum values with individual data points. **G** Representative confocal images of PyNs in layer V of DCZ-treated wild-type (WT), *15q dup*, *Rbp4-Cre*, and *15q dup*; *Rbp4-Cre* mice. Scale bar = 10µm. **H**, **I** Quantification of AIS length (**H**) and cumulative frequency of AIS length (**I**) in layer V PyNs for each genotype. Values are presented as medians with 25–75% intervals, and the error bars indicate the minimum and maximum values with individual data points. *P<0.05 **P<0.01 analyzed with one-way analysis of variance (ANOVA) followed by post-hoc Tukey’s test (n=5 mice per group; >50 AIS per mouse). **J**, **K** Quantification of AIS length (**J**) and cumulative frequency of AIS length (**K**) of mCherry-negative PyNs located outside the DREADD-targeted areas of the mPFC in each genotype. Values are presented as medians with 25–75% intervals, and the error bars indicate the minimum-to-maximum values with individual data points. *P<0.05, **P<0.01 analyzed with unpaired t-test for E, Mann–Whitney U test for F, and one-way analysis of variance (ANOVA) followed by post-hoc Tukey’s (**H**, **J**, **K**) (*n*=5; >50 AIS per mouse). A summary of all statistical values is provided in Table S9.

We investigated whether abnormal AIS length could be rescued by neuronal activation using the DREADD system. We used a sophisticated intersectional strategy combining the Con/Fon system with a retrogradely transported adeno-associated virus vector (AAV-retro-Flpo) in *Rbp4-Cre* transgenic mice (Fig. 4A, B) to specifically target mPFC layer V PyNs projecting to the DRN [32], ensuring that the excitatory Gq-DREADD receptor was expressed only in layer V PyNs that project to the DRN (Fig. 4C). Mice received a single intraperitoneal injection of DCZ four weeks after viral injection. One hour after injection, we confirmed successful neuronal activation via c-Fos expression (Fig. 4D) and quantified AIS length. This single activation was sufficient to induce a significant rescue (re-elongation) of the AIS length in the targeted PyNs of *15q dup; Rbp4-Cre* mice compared with saline-treated controls (*n*=5 mice per group; >50 AIS per mouse; Fig. 4E). This structural rescue was confirmed by a significant rightward shift in the cumulative frequency distribution (Fig. 4F).

We compared AIS lengths across four genotypes receiving DCZ to confirm that this rescue was specific to the targeted circuit activation and not a non-specific effect of DCZ or DREADD expression (*n*=5 mice per group; >50 AIS per mouse; Fig. 4G–I). A significant rescue effect was observed only in the *15q dup*; *Rbp4-Cre* mice. The AIS length in *15q dup* control mice remained short, demonstrating that the rescue required specific DREADD activation in the target population (Fig. 4H, I).

Finally, we compared the AIS lengths of DREADD-expressing (mCherry^+^) and non-expressing (mCherry^–^) PyNs within the same *15q dup; Rbp4-Cre* animals to test if this effect was cell-autonomous. The AIS length was significantly rescued only in the mCherry^+^ PyNs. Neighboring mCherry^–^ PyNs, which presumably included the affected NAcc- and LHb-projecting cells, remained in their shortened, pathological state (*n*=5 mice per group; >50 AIS per mouse; Fig. 4J, K).

These results indicated that DREADD-mediated restoration of neuronal activity in the mPFC–DRN circuit effectively and cell-autonomously rescued the abnormal AIS shortening. All statistical data for these comparisons are detailed in Table S9.

### Rescue of aberrant AIS in the mPFC–DRN circuit ameliorates ASD-like behavioral deficits

**Fig. 5:**
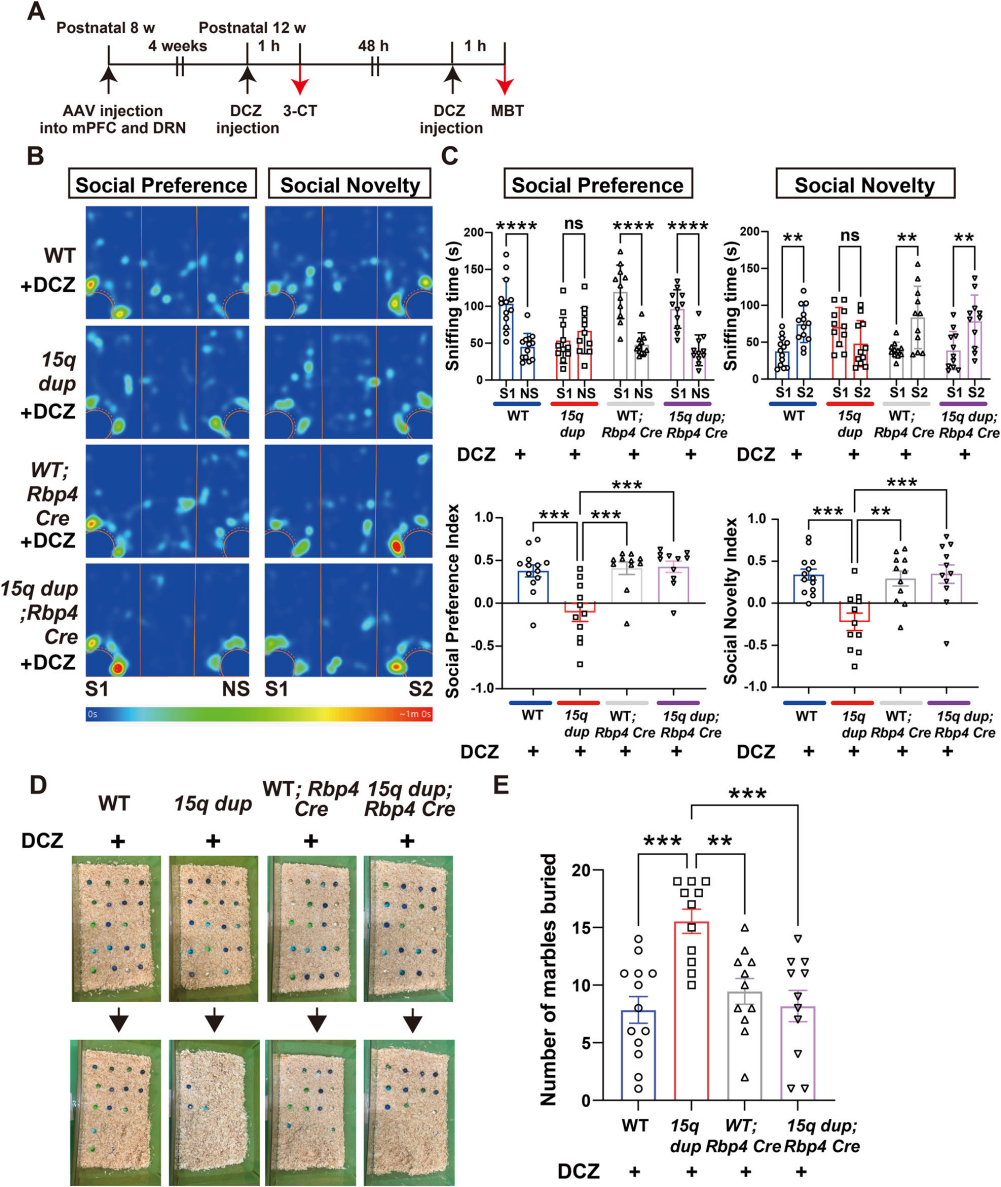
Activation of pyramidal neurons (PyNs) in the medial prefrontal cortex (mPFC) projecting to the dorsal raphe nucleus (DRN) can rescue social behavior in *15q dup*; *Rbp4-Cre* mice using the designer receptors exclusively activated by designer drugs (DREADD) system. **A** Schematic representation of the experimental timeline. **B** Representative heat maps from the three-chamber test (3-CT) showing social preference and social novelty behavior in wild-type (WT), *15q dup*, *Rbp4-Cre*, and *15q dup*; *Rbp4-Cre* mice. Mice were injected with an adeno-associated virus (AAV) vector encoding Con/Fon DREADD Gq-mCherry into the mPFC and AAV retro-Flpo combined with 0.1% cholera toxin B (CTB) into the DRN. **C** Quantification of social preference and novelty in the 3-CT test. The upper panels display the sniffing time, and the lower panels show the social preference and novelty index. Data are presented as mean±standard error of the mean (SEM). ns, not significant; social preference and social novelty ****P<0.0001, **P<0.01 analyzed with two-way analysis of variance (ANOVA) followed by post-hoc Sidak’s test (*n*=11 mice per group); social preference and novelty indices ***P<0.001, **P<0.01 analyzed with one-way ANOVA followed by post-hoc Tukey’s test (*n*=11 mice per group). **D** Representative images of marble-burying behavior. The upper panels show the field before the test, and the lower panels show the results after the test in WT, *15q dup*, *Rbp4-Cre*, and *15q dup*; *Rbp4-Cre* mice. **E** Quantification of the number of marbles buried. ***P<0.001, **P<0.01, analyzed with one-way ANOVA followed by post-hoc Tukey’s test (*n*=11 mice per group). Data are presented as mean±SEM. A summary of all statistical values is provided in Table S10.

Finally, we investigated whether the successful DREADD-mediated structural rescue could ameliorate behavioral abnormalities in *15q dup* mice [22, 23]. We used the same four-genotype cohort of mice from the AIS rescue experiment (WT, *15q dup*, *Rbp4-Cre*, and *15q dup; Rbp4-Cre*). We assessed social and repetitive behaviors using the 3-CT and MBT. Behavioral testing was initiated 1h after DCZ injection for the 3-CT; a second DCZ injection was administered 48h later, 1h before the MBT (Fig. 5A). In the 3-CT, *15q dup* control mice exhibited the expected ASD-like deficits, showing significant impairments in social preference (time spent with a social stimulus S1 vs. a non-social object NS) and social novelty (time spent with a novel mouse S2 vs. the familiar S1). A single DCZ treatment dramatically rescued both deficits, specifically in the *15q dup; Rbp4-Cre* mice. The targeted intervention restored both social preference and social novelty indices to levels indistinguishable from WT controls (*n*=11 mice per group; Fig. 5B, C).

Following the 3-CT, we assessed repetitive behavior using the MBT. *15q dup* control mice buried significantly more marbles than WT controls, indicative of increased repetitive/anxiety-like behavior. This deficit was completely rescued in the DCZ-treated *15q dup; Rbp4-Cre* mice, which buried a number of marbles comparable to WT levels (*n*=11 mice per group; Fig. 5D, E).

Consequently, we concluded that the amelioration of AIS length in the mPFC–DRN circuit is sufficient to rescue core behavioral symptoms associated with the *15q dup* ASD model. All statistical details for these behavioral tests, including ANOVA and post-hoc analyses, are available in Table S10.

## Discussion

In this study, we demonstrated that a clinically relevant mouse model of ASD exhibits projection-specific shortening of the AIS in cortical PyNs, resulting in reduced neuronal excitability and apparent impairment of homeostatic plasticity. Crucially, we showed that these structural and functional deficits are not permanent but represent maladaptive, yet reversible forms of plasticity. Targeted chemogenetic activation of a single dysfunctional circuit, the mPFC–DRN pathway, was sufficient to restore normal AIS structure and rescue core ASD-like behavioral abnormalities. Notably, the observed shortening of the AIS was not due to diminished presynaptic input or impaired local network connectivity. A quantitative analysis of Synaptophysin-1-positive presynaptic boutons across various cortical regions in layers II/III and V showed no genotype-dependent differences (Figs. S2A and S2B). Although our analysis does not directly evaluate functional presynaptic parameters, the maintained bouton density demonstrates that the AIS phenotype cannot be attributed to a loss of presynaptic structural input.

Our findings revealed a paradoxical physiological state in the PrL of *15q dup* mice, wherein layer V PyNs with increased excitatory synaptic inputs exhibited decreased action potential outputs (Fig. 2). While this phenomenon may be partly attributable to alterations in local inhibitory circuits and 5-HT receptor expression patterns [40], it highlights the critical role of homeostatic adaptation, a mechanism by which neurons adjust their intrinsic excitability to stabilize network activity [41]. Such adaptations can occur in multiple cellular compartments, including AIS [41].

A recent study showed that this AIS plasticity is not merely phenomenological but is driven by concrete molecular mechanisms [2, 42]. Increased network activity or direct N-methyl-D-aspartate receptor (NMDAR) activation can trigger rapid AIS shortening within minutes to hours, which increases the AP threshold and dampens neuronal excitability. A groundbreaking study by Fréal et al. demonstrated that this rapid shortening is not caused by scaffold degradation but by the clathrin-mediated endocytosis of Nav channels (specifically Nav1.2) from the membrane surface [43]. This finding provides a direct molecular link between synaptic input (NMDAR activity) and AIS structure/function. Critically, this endocytic mechanism implies that the process is reversible, as internalized channels can be stored in a “readily releasable recycling pool” rather than being immediately degraded [43].

Previous studies have shown that *Tau* mutations associated with frontotemporal dementia impair AIS plasticity through an EB3-dependent mechanism [17], resulting in a pathologically static AIS. In contrast to these irreversible defects, our data indicate that AIS shortening in *15q dup* mice was not reversed by high potassium treatment. However, this lack of plasticity is not due to EB3 dysfunction. Immunostaining revealed no significant differences in EB3 or Ankyrin-G distribution between wild-type and *15q dup* mice, suggesting that EB3-mediated cytoskeletal abnormalities were not involved. Importantly, the restoration of AIS length and function through DREADD-mediated activation provides the most compelling evidence that AIS alterations in *15q dup* mice are not fixed structural defects but rather reversible, activity-dependent, and manipulatable changes.

We argue this apparent lack of plasticity in response to high potassium is not an impairment of the underlying molecular machinery; rather, it is a “floor effect.” Our in vivo data show that the AIS in *15q dup* PrL neurons is already significantly shortened at baseline (Fig. 1B), likely as a homeostatic compensation for the chronic hyperexcitability (Fig. 2E). These neurons are already in a maximally shortened adaptive state; therefore, in vitro KCl depolarization (a shortening stimulus) cannot elicit a further detectable change. This interpretation is strongly supported by our DREADD rescue experiment (Fig. 4). The successful re-elongation of the AIS in vivo demonstrates that these neurons fully retain their capacity for bidirectional plasticity, confirming that the baseline state was a reversible “floor effect,” not a permanent pathological defect.

This “floor effect” model, driven by chronic excitatory input, integrates well with known 5-HT deficits in this model [23]. In the PrL, reduced 5-HT inhibition of inhibitory interneurons may shift the E/I balance, leading to AIS shortening and a hypoexcitable state [43]. Critically, these structural changes in the AIS were not uniform; instead, they showed a striking dependence on neuron-specific projection targets (Fig. 3). This finding suggests that these are not random cellular defects but rather finely tuned, circuit-specific regulatory mechanisms. This aligns with previous work showing that even subtle AIS remodeling can influence spike dynamics [44], indicating a novel mechanism by which circuit-specific dysfunction can arise in neurodevelopmental disorders.

Building on this mechanistic insight into AIS plasticity, we further explored the neurocircuit specificity of AIS alterations in defined projection pathways, as accumulating evidence indicates that the mPFC controls social behaviors in a projection target-specific manner [45, 46]. In the *15q dup* ASD model, we detected abnormal AIS morphology in PyNs projecting from the PrL to the NAcc, LHb, and DRN but not to the VTA, CPVM, or BLA. In our study, chemogenetic activation of mPFC layer V PyNs projecting to the DRN using the DREADD and Con/Fon systems rescued social and repetitive behavioral deficits in *15q dup* mice and restored AIS length in these projection-specific neurons (Figs. 4, 5). Notably, this manipulation did not produce a significant change in normal AIS length, suggesting that it selectively normalized the abnormally shortened AIS rather than altering the AIS length under baseline conditions. These findings suggest that selective restoration of AIS plasticity in the mPFC–DRN pathway can normalize excitability in circuits relevant to social behavior, offering a potential therapeutic approach for treating ASD.

The mPFC–DRN pathway has emerged as a key circuit for regulating socioaffective behaviors [46, 47]. The DRN, a primary source of 5-HT in the forebrain, receives monosynaptic inputs from the mPFC [48, 49]. Previous studies have shown that these projections can synapse onto GABAergic and 5-HT neurons in a topographically distinct manner [48, 49]. Functionally, this circuit appears to be involved in various dimensions of stress-related and social behaviors. For instance, activity in the mPFC-DRN circuit has been shown to mediate the transition between active escape and behavioral despair [50], contribute to resistance against acute social defeat [51] and promote the acquisition of social avoidance [48]. Together, these findings suggest that mPFC–DRN connectivity serves as a dynamic node for regulating social engagement, emotional reactivity, and stress-related responses.

Our finding that AIS abnormalities in layer V PyNs in the mPFC projecting specifically to the DRN are associated with autism-like social deficits further supports the critical role of this pathway in social behavior. The ability to rescue both AIS plasticity and social dysfunction via selective chemogenetic activation of the mPFC–DRN circuit emphasizes that specific long-range projections, not just regional activity, are key determinants of behavioral outcomes. These results advance previous evidence by identifying AIS structural modulation as a cellular mechanism linking projection-specific excitability to behavioral phenotypes. Therefore, circuit-selective restoration of AIS plasticity may offer a novel avenue for therapeutic intervention in neurodevelopmental disorders, such as ASD. Future therapeutic strategies may include pharmacological agents targeting AIS-associated proteins and circuit-based interventions, such as transcranial magnetic stimulation, aimed at normalizing activity in specific pathways, such as the mPFC–DRN circuit identified in this study.

Finally, we acknowledge several important limitations in the present study. First, while we primarily evaluated Ankyrin-G and Nav channels, a comprehensive understanding of AIS structural remodeling will require future investigations incorporating other scaffolding proteins, such as β4-spectrin. Second, our in vitro plasticity assays relied on fixed-tissue snapshots; utilizing live-cell imaging approaches would allow for a more direct and accurate assessment of real-time AIS plasticity dynamics. Third, while we observed population-level decreases in neuronal excitability in the PrL, the structural AIS shortening was highly projection-specific. Therefore, future studies utilizing targeted patch-clamp recordings on retrogradely labeled neurons are essential to directly demonstrate the precise functional impact of these projection-specific AIS alterations at the single-cell level.

In summary, our AIS analysis provides insights into the structural integrity of neurons and contributes to a comprehensive perspective on the neural network dysfunction characteristic of neuropsychiatric conditions, including ASD.

## Supplementary information

Supplementary Information

Original Data

## Data Availability

The data that support the findings of this study are available within the article and its Supplementary Information files. Source data are provided with this paper.
