# Supplementary material for: Restoration of axon initial segment plasticity via chemogenetic activation rescues autism-related behaviors
Source: Cell Death Dis. 2026 May 19;17(1):634. doi: 10.1038/s41419-026-08873-0 (PMC13357743; doi:10.1038/s41419-026-08873-0)
Supplement: Supplementary file 1 — Supplementary Information [file 41419_2026_8873_MOESM1_ESM.pdf]

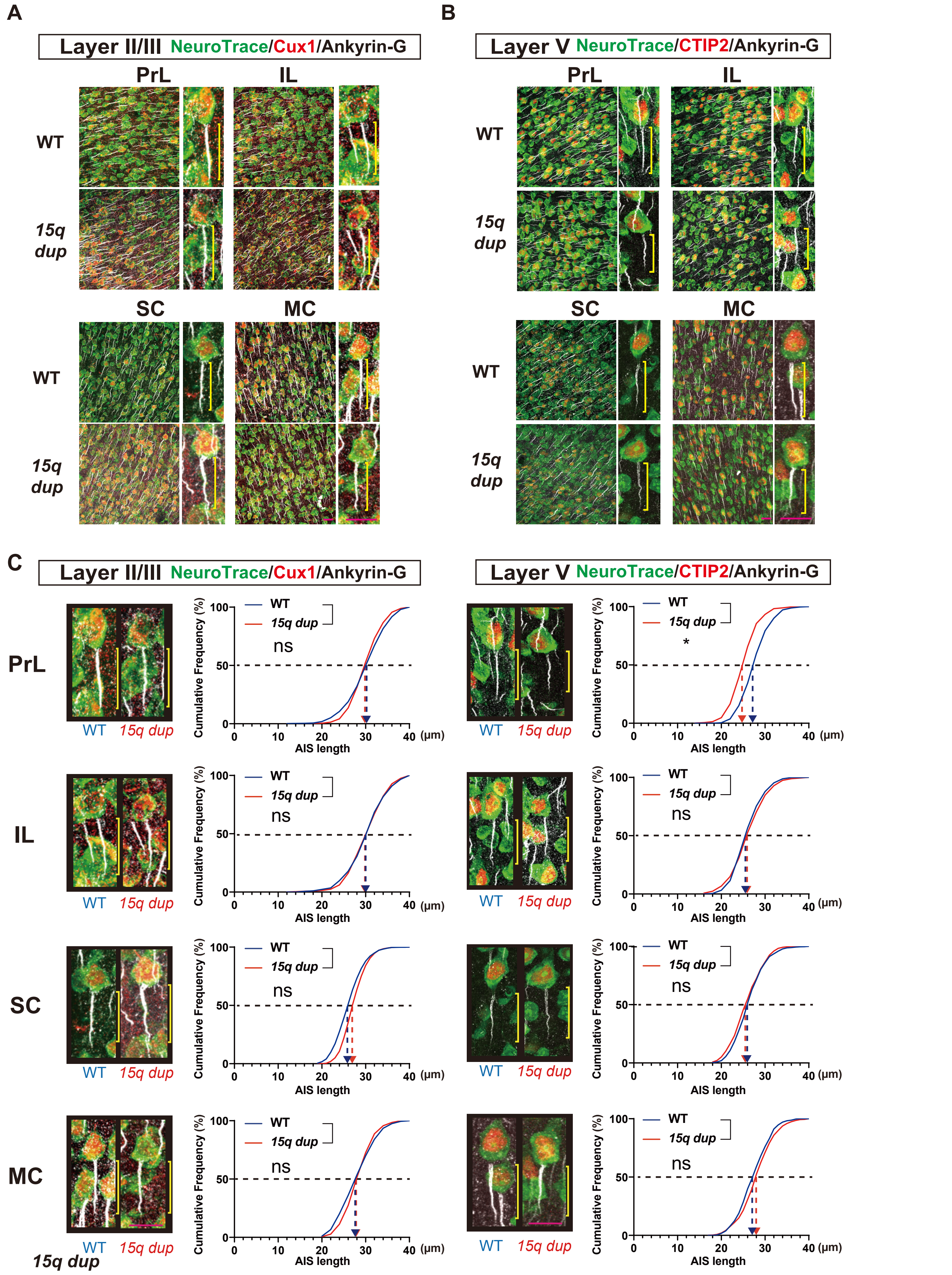

Figure S1. Axon initial segment (AIS) length in cortical pyramidal neurons of *15q* dup mice, related to Figure 1
(A) Representative confocal images of layer II/III pyramidal neurons (PyNs) in the prelimbic (PrL), infralimbic (IL), somatosensory (SC), and motor (MC) cortices of wild-type (WT) and *15q dup* mice. Neurons are stained for Cux1 (red; layer II/III marker), Ankyrin-G (white; AIS marker), and NeuroTrace® (green; Nissl stain). **(B)** Representative confocal images of layer V PyNs in the same cortical regions. Neurons are stained for CTIP2 (red; layer V marker), Ankyrin-G (white), and NeuroTrace® (green). **(C)** Cumulative frequency distributions of AIS length in layer II/III and layer V PyNs for each cortical region. The arrows indicate the AIS length at 50% of the cumulative frequency distribution curve. Statistical analysis was performed using the Mann–Whitney U test. P < 0.05; ns, not significant. Yellow bars in the images indicate the measured AIS length for each neuron. Scale bar = 10 μm. A summary of all statistical values is provided in Table S2.

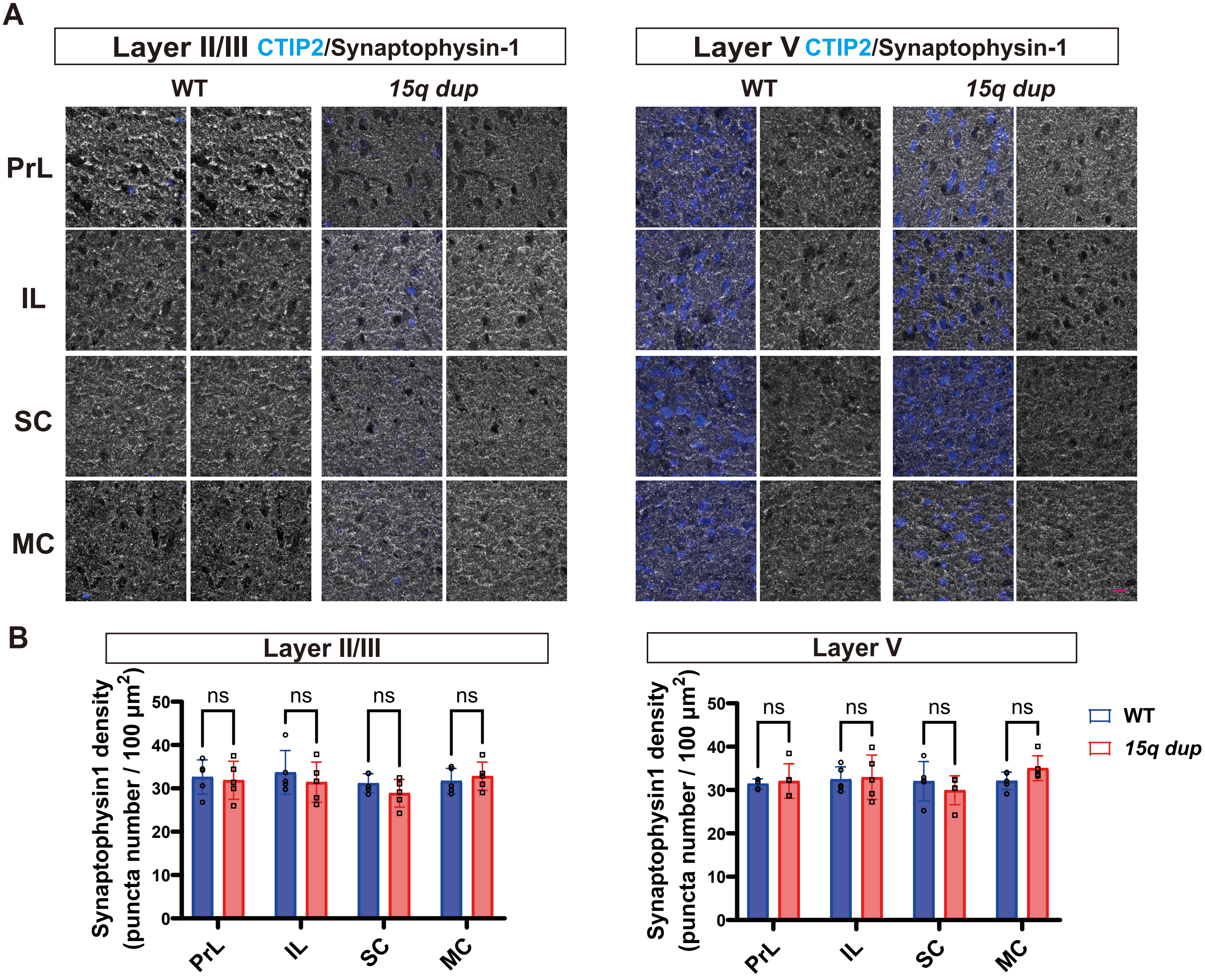

Figure S2. The quantification of presynaptic puncta.

(A) Representative confocal images of layer II/III and layer V PyNs in the PrL, IL, SC, and MC cortices of WT and *15q dup* mice. Neurons were stained for CTIP2 (blue; a marker for layer V) and Synaptophysin-1 (white; presynaptic marker). Scale bar = 10 µm. **(B)** Quantification of Synaptophysin1-positive presynaptic puncta in layer II/III and layer V of each cortical region. All values represent means ± SEM (n = 5 mice per group). Two-way analysis of variance (ANOVA) Sidak's post-hoc test: all ns. A summary of all statistical values is provided in Table S4.

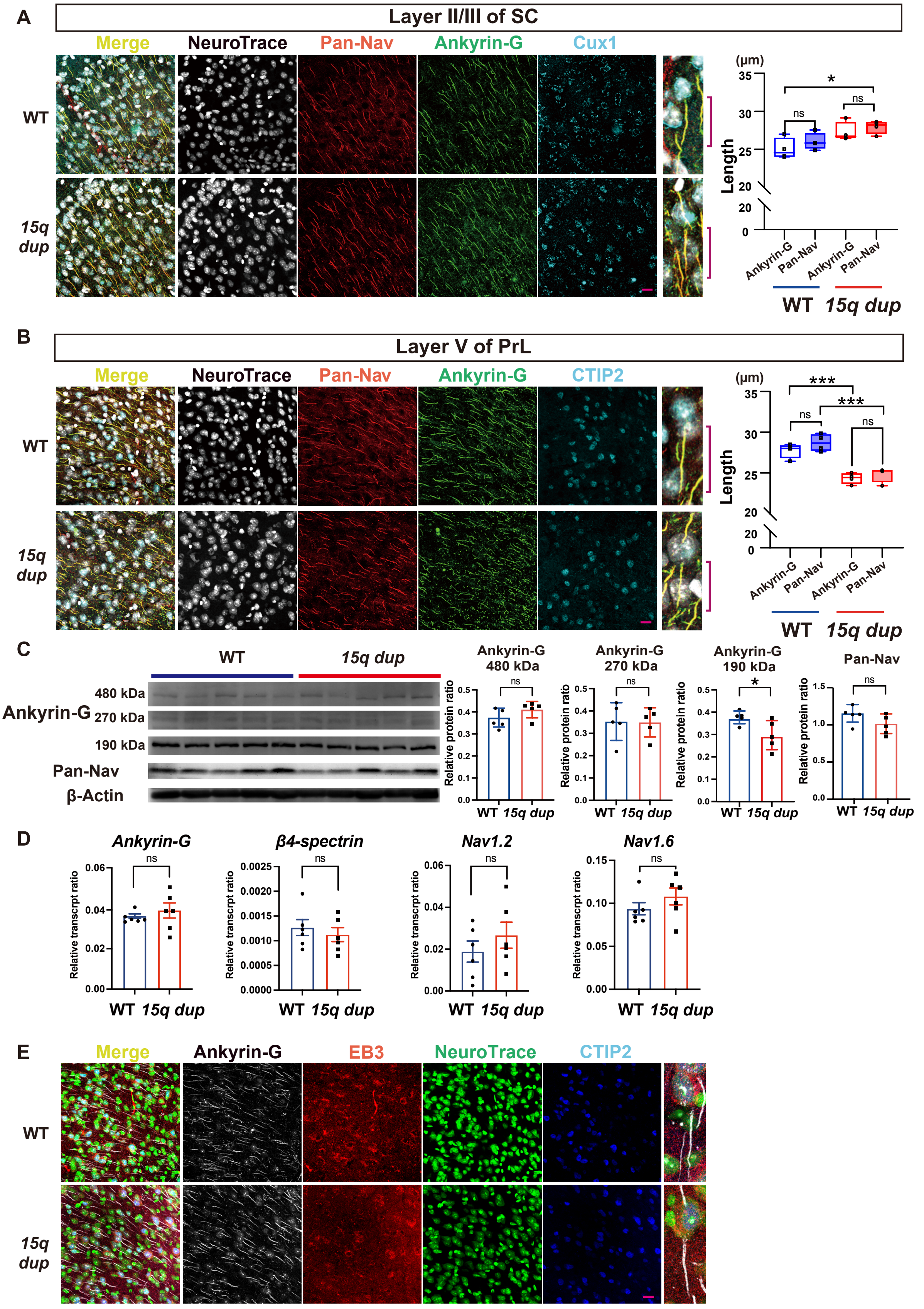

Figure S3. Biochemical analysis of the axon initial segment (AIS) components in *15q dup* mice, related to Figure 1

(A) Representative confocal images and quantification of Ankyrin-G and Pan-voltage-gated sodium (Pan-Nav) channel cluster lengths in Layer II/III pyramidal neurons (PyNs) of the somatosensory cortex (SC). Neurons are stained for Cux1 (cyan, layer II/III marker), Pan-Nav (red), Ankyrin-G (green), and NeuroTrace® (white). (B) Representative confocal images and quantification for layer V PyNs of the prelimbic cortex (PrL). Neurons are stained for CTIP2 (cyan, layer V marker), Pan-Nav(red), Ankyrin-G (green), and NeuroTrace® (white). (C) Representative immunoblots and quantification of Ankyrin-G isoforms (480, 270, and 190 kDa) and Pan-Nav in mPFC homogenates from wild-type (WT) and 15q dup mice. Protein levels are normalized to β-actin.

(D) Quantitative real-time polymerase chain reaction (PCR) analysis of *Ankyrin-G, β4-spectrin, Nav1.2*, and *Nav1.6* transcript levels in the whole cortex, normalized to β-actin. (E) Representative confocal images of layer V PyNs in the PrL, stained for end-binding protein 3 (EB3; red), Ankyrin-G (white), CTIP2 (blue), and NeuroTrace®(green). For box plots in (A) and (B), data are presented as medians with 25–75% and minimum-to-maximum whiskers, with individual data points shown (n = 4 mice per group). For scatter plots in (C) and (D), data are presented as mean ± standard error of the mean (SEM) with individual data points shown (n = 6 mice per group). Statistical analysis was performed using a one-way ANOVA with Tukey's post-hoc test for (A, B) or a two-tailed unpaired Student's t-test for (C, D). *$P < 0.05$, ***$P < 0.001$; ns, not significant. Colored vertical bars alongside the magnified neurons in (A) and (B) indicate the measured length of the protein clusters. Scale bars = 10 μm. A summary of all statistical values is provided in Table S5.

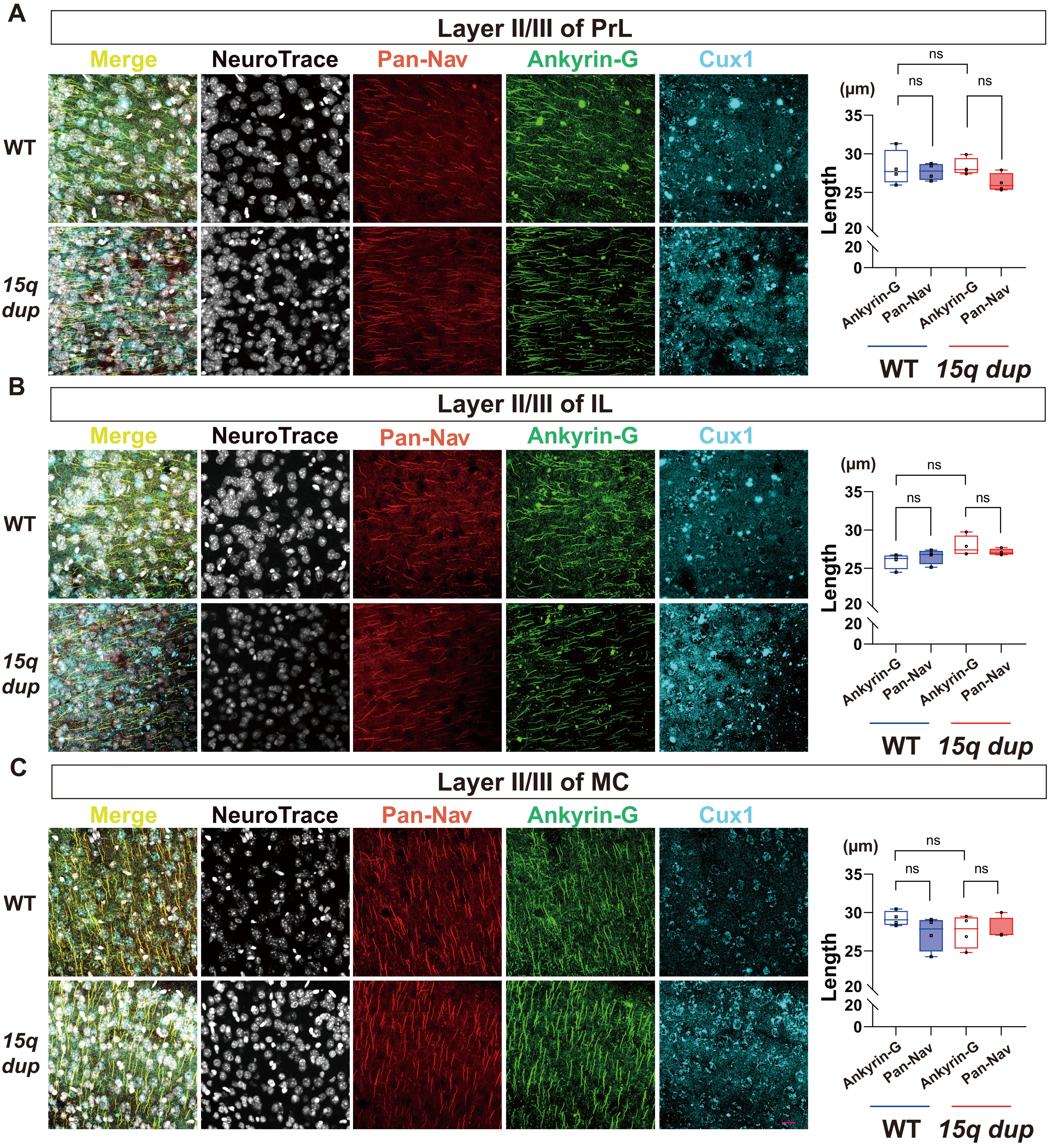

Figure S4. Co-localization of Ankyrin-G and Pan-Nav channels in layer II/III pyramidal neurons, related to Figure S3A

Representative confocal images and quantification of Ankyrin-G and Pan-voltage-gated sodium (Pan-Nav) channel cluster lengths in layer II/III pyramidal neurons (PyNs) of the (A) prelimbic cortex (PrL), (B) infralimbic cortex (IL), and (C) motor cortex (MC) in wild-type (WT) and *15q dup* mice. Neurons are stained for Cux1 (cyan), Pan-Nav (red), Ankyrin-G (green), and NeuroTrace® (white).

Data are presented as box-and-whisker plots showing individual data points. The box extends from the 25–75% the central line indicates the median, and whiskers show the minimum-to-maximum values with individual data points. Statistical analysis was performed using a one-way ANOVA with Tukey's post-hoc test (n = 4 mice per group; >50 AIS per mouse). ns, not significant. Scale bar = 10 μm. A summary of all statistical values is provided in Table S6.

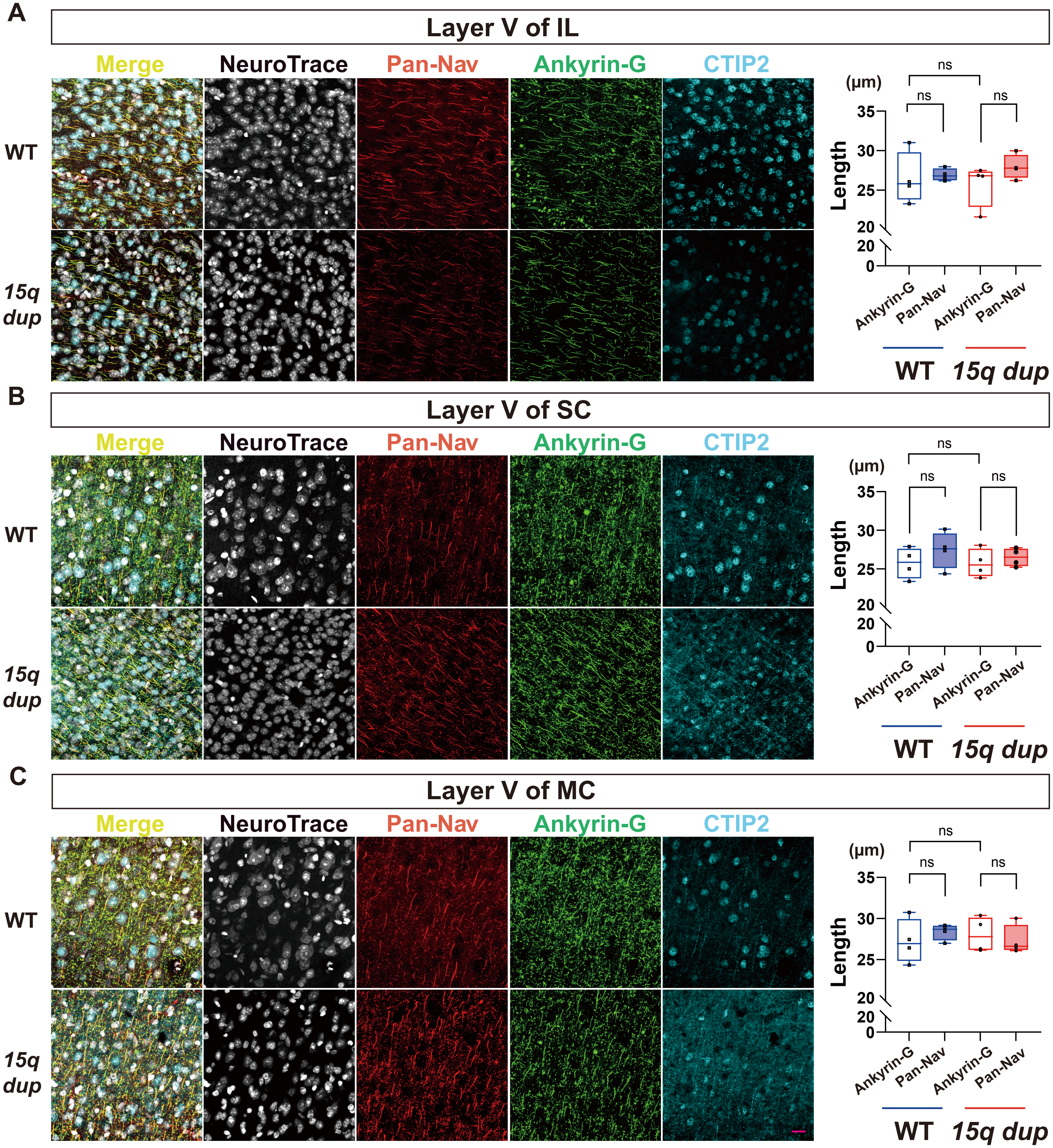

Figure S5. Co-localization of Ankyrin-G and Pan-Nav channels in layer V pyramidal neurons, related to Figure S3B
Representative confocal images and quantification of Ankyrin-G and Pan-voltage-gated sodium (Pan-Nav) channel cluster lengths in layer V pyramidal neurons (PyNs) of the (A) infralimbic cortex (IL), (B) somatosensory cortex (SC), and (C) motor cortex (MC) in wild-type (WT) and *15q dup* mice. Neurons are stained for CTIP2 (cyan, layer V marker), Pan-Nav (red), Ankyrin-G (green), and NeuroTrace® (white).
Data are presented as box-and-whisker plots showing individual data points. The box extends from the 25–75% the central line indicates the median, and whiskers show the minimum-to-maximum values with individual data points. Statistical analysis was performed using a one-way ANOVA with Tukey's post-hoc test (n = 4 mice per group; >50 AIS per mouse). ns, not significant. Scale bar = 10 μm. A summary of all statistical values is provided in Table S7.

Table S1: Statistical details of axon initial segment (AIS) structure and plasticity in wild-type (WT) and *15q dup* mice.

Figure Panel	Description	Sample Size (n)	Statistical Test	Data (Median [25–75%]) & Statistical Details
**Fig. 1B (L II/III)**	AIS Length<br>(Layer II/III)	n = 7 mice/group<br>>50 AIS/mouse	Two-way ANOVA,<br>Sidak's post-hoc	Interaction (Region × Genotype): F(3, 48) = 0.2909, P = 0.8318<br>Main effect (Region): F(3, 48) = 6.057, P = 0.0014<br>Main effect (Genotype): F(1, 48) = 0.07077, P = 0.7914<br>Post-hoc:<br>PrL (WT): 30.60 (27.42-32.24) µm vs PrL (*15q dup*): 29.43 (29.14-32.78) µm, P = 0.9652<br>IL (WT): 30.93 (26.46-32.05) µm vs IL (*15q dup*): 28.16 (27.17-33.18) µm, P = 0.7312<br>SC (WT): 26.98 (25.13-27.77) µm vs SC (*15q dup*): 27.61 (27.14-27.71) µm, P = 0.3705<br>MC (WT): 28.99 (27.19-31.03) µm vs MC (*15q dup*): 28.78 (27.67-29.64) µm, P = 0.9443
**Fig. 1B (L V)**	AIS Length<br>(Layer V)	n = 7 mice/group<br>>50 AIS/mouse	Two-way ANOVA,<br>Sidak's post-hoc	Interaction (Region × Genotype): F(3, 48) = 2.054, P = 0.1188.<br>Main effect (Region): F(3, 48) = 1.843, P = 0.1520<br>Main effect (Genotype): F(1, 48) = 0.7722, P = 0.3839<br>Post-hoc:<br>**PrL (WT): 28.21 (26.12-30.38) µm vs PrL (*15q dup*): 25.75 (23.76-27.77) µm, P = 0.0198 (*)**<br>IL (WT): 26.37 (25.87-27.79) µm vs IL (*15q dup*): 27.32 (24.37-30.12) , P = 0.6187<br>SC (WT): 26.72 (26.02-27.20) µm vs SC (*15q dup*): 26.73 (25.17-27.21) µm, P = 0.5636<br>MC (WT): 28.54 (26.34-29.07) µm vs MC (*15q dup*): 28.25 (26.98-29.57) µm, P = 0.4671
**Fig. 1D**	AIS Plasticity<br>(PrL, KCl)	n = 4–6<br>slices/group<br>>50 AIS/mouse	Two-way ANOVA,<br>Tukey's post-hoc	Interaction (Time × Genotype): F(2, 22) = 3.687, P = 0.0416<br>Main effect (Time): F(2, 22) = 5.275, P = 0.0134<br>Main effect (Genotype): F(1, 22) = 13.66, P = 0.0013<br>WT Data: 0h: 28.09 (27.17-32-05) µm; 1h: 25.55 (23.61-26.79) µm; 3h: 28.72 (27.48-31.52) µm<br>15q dup Data: 0h: 26.92 (25.91-27.09) µm; 1h: 26.47 (23.16-26.66) µm; 3h: 24.57 (22.45-24.57) µm<br>**Post-hoc (WT): 0h-1h P = 0.0023 (**); 0h-3h P = 0.9997 (ns); 1h-3h P = 0.0038 (**)**<br>Post-hoc (*15q dup*): 0h-1h P = 0.2928 (ns); 0h-3h P = 0.1138 (ns); 1h-3h P = 0.5357 (ns)<br>**Post-hoc (WT vs *15q dup*): 0h-0h P = 0.0453 (*)**; 1h-1h P = 0.8060 (ns); **3h-3h P = 0.0011 (**)**
**Fig. 1F**	AIS Plasticity<br>(IL, KCl)	n = 4–6<br>slices/group<br>>50 AIS/mouse	Two-way ANOVA,<br>Tukey's post-hoc	Interaction (Time × Genotype): F(2, 22) = 1.377, P = 0.2731<br>Main effect (Time): F(2, 22) = 6.962, P = 0.0045<br>Main effect (Genotype): F(1, 22) = 0.9135, P = 0.3496<br>WT Data: 0h: 26.28 (25.78-29.04) µm; 1h: 23.03 (21.46-24.48) µm; 3h: 27.11 (24.70-28.42) µm<br>15q dup Data: 0h: 25.60 (24.57-26.87) µm; 1h: 23.29 (22.76-25.62) µm; 3h: 25.97 (22.36-27.53) µm<br>**Post-hoc (WT): 0h-1h P = 0.0015 (**)**; 0h-3h P = 0.7182 (ns); **1h-3h P = 0.0062 (**)**<br>Post-hoc (*15q dup*): 0h-1h P = 0.1966 (ns); 0h-3h P = 0.7684 (ns); 1h-3h P = 0.3200 (ns)<br>Post-hoc (WT vs *15q dup*): 0h-0h P = 0.2388 (ns); 1h-1h P = 0.4240 (ns); 3h-3h P = 0.2833 (ns)

Table S2. Statistical details of axon initial segment (AIS) length in cortical pyramidal neurons of wild-type (WT) and *15q dup* mice.

Figure Panel	Description	Sample Size (n)	Statistical Test	Data (Mean ± SEM) & Statistical Details
**Fig. S1C (L II/III)**	AIS Length (Layer II/III, Cumulative frequency distribution)	(n=7 mice)	Mann-Whitney U test	PrL: U=20, 30.56 ± 1.193 μm vs PrL (*15q dup*): 30.62 ± 0.8110 μm, P = 0.620 (ns)<br>IL: U=23, 30.33 ± 1.408 μm vs IL (*15q dup*): 29.88 ± 1.164 μm,P = 0.8747 (ns)<br>SC: U=14, 26.47 ± 0.4864 μm vs SC (*15q dup*): 27.68 ± 0.2659 μm, P = 0.1952 (ns)<br>MC: U=24, 28.61 ± 0.8776 μm vs MC (*15q dup*): 28.51 ± 0.5293 μm, P > 0.9999 (ns)
**Fig. S1C (L V)**	AIS Length (Layer V, Cumulative frequency distribution)	(n=7 mice)	Mann-Whitney U test	**PrL: U=6, 28.57 ± 0.4889 μm vs PrL (*15q dup*): 26.13 ± 0.6513 μm, P = 0.0175 (*)**<br>IL: U=22, 26.86 ± 0.7923 μm vs IL (*15q dup*): 27.37 ± 1.108 μm, P = 0.8048 (ns)<br>SC: U=23.5, 26.82 ± 0.5559 μm vs SC (*15q dup*): 26.23 ± 0.5137 μm, P = 0.9289 (ns)<br>MC: U=20, 27.81 ± 0.5191 μm vs MC (*15q dup*): 28.55 ± 0.8460 μm, P = 0.6200 (ns)

Table S3. Statistical details of electrophysiological properties in layer V pyramidal neurons of wild-type (WT) and 15q dup mice.

	PrL			IL		
	F	*degrees of freedom*	p-value	F	*degrees of freedom*	p-value
Interaction	F(11,418)	1.812	0.05*	F(11,418)	0.6302	0.8032
Time	F(11,418)	90.15	< 0.0001	F(11,418)	99.33	< 0.0001
Genotype	F(1,38)	0.7456	0.3933	F(1,38)	0.7379	0.3957

	PrL			IL		
	WT	*15q dup*	p-value	WT	*15q dup*	p-value
	N=20/4 mice	N=20/4mice		N=20/4 mice	N=20/4mice	
Action potential frequency (Hz)						
-50 pA	0	0	>0.9999	0	0	>0.9999
0 pA	0	0	>0.9999	0	0	>0.9999
50 pA	0.9 (± 0.4224)	0	0.6723	0.6 (± 0.3584)	0.2 (± 0.2000)	0.8773
100 pA	5.7 (± 0.9434)	2.6 (± 0.6000)	0.1455	4.6 (± 0.8838)	4.1 (± 0.9344)	0.8469
150 pA	10.6 (± 1.027)	6.6 (± 1.037)	0.0606	9.8 (± 1.264)	8.7 (± 1.316)	0.6711
200 pA	14.3 (± 1.108)	10.1 (± 1.261)	**0.0488***	13.8 (± 1.528)	12.3 (± 1.409)	0.5626
250 pA	17.1 (± 1.294)	12.9 (± 1.380)	**0.0488***	17.1 (± 1.720)	14.7 (± 1.712)	0.3544
300 pA	17.1 (± 1.454)	15.1 (± 1.566)	0.3474	19.4 (± 2.052)	16.9 (± 2.034)	0.3347
350 pA	17 (± 1.928)	16.8 (± 1.670)	0.9251	20.7 (± 2.300)	18.5 (± 2.397)	0.3959
400 pA	16.8 (± 2.259)	17.5 (± 2.010)	0.7421	21.8 (± 2.659)	19.2 (± 2.455)	0.3157
Current Threshold (pA)						
	329.5 (± 22.69)	368.5 (± 19.09)	t=1.315, df=38 P=0.1963	281.5 (± 16.15)	292.5 (± 14.12)	t=0.5128, df=38 P=0.611
Voltage Threshold (mV)						
	-37.95 (± 2.208)	-41.5 (± 1.728)	t=1.265, df=38 P=0.2135	-37.20 (± 1.569)	-38.65 (± 1.787)	t=0.6112, df=38 P=0.5447
Spontaneous Excitatory Postsynaptic Currents (sEPSC) Frequency (Hz)						
	2.752 (± 0.3499)	5.333 (± 1.095)	**t=2.244, df=38 P=0.0307***	4.473 (± 0.8842)	3.5 (± 0.9234)	t=0.7601, df=38 P=0.4519
sEPSC Amplitudes (pA)						
	12.49 (± 0.4643)	15.66 (± 1.991)	t=1.552, df=38 P=0.129	14.52 (± 1.100)	13.71 (± 0.9234)	t=0.5678, df=38 P=0.5735
f' (Max) (Hz/pA)						
	0.07267 (± 0.006789)	0.04699 (± 0.004247)	**t=3.207, df=38 P=0.0027****	0.06535 (± 0.007239)	0.05833 (± 0.006909)	t=0.7010, df=38 P=0.4876
I at f' (Max) (pA)						
	127.5 (± 7.637)	162.5 (± 13.99)	**t=2.193, df=38 P=0.0345***	155 (± 18.10)	140 (± 8.584)	t=0.7488, df=38 P=0.4586
Resting membrane potential (RMP) (mV)						
	-77.72 (± 1.997)	-76.12 (± 2.058)	t=0.5574, df=38 P=0.5805	-73.34 (± 1.555)	-76.87 (± 2.021)	t=1.381, df=38 P=0.1752

Table S4. Statistical details of presynaptic synaptophysin-1 puncta density in cortical pyramidal neurons of wild-type (WT) and *15q dup* mice.

Figure Panel	Description	Sample Size (n)	Statistical Test	Data (Mean ± SEM) & Statistical Details
**Fig. S2B (L II/III)**	The density of Synaptophysin-1-positive Puncta (Layer II/III)<br>Unit = Puncta/100 μm^2	n = 5 mice/group	Two-way ANOVA, Sidak's post-hoc	Interaction (Region × Genotype): F(3,32) = 0.4478, P = 0.7205<br>Main effect (Region): F(3,32) = 0.9502, P = 0.4280<br>Main effect (Genotype): F(1,32) = 0.7490, P = 0.3932<br>Post-hoc:<br>PrL (WT): 32.61 ± 1.774 vs PrL (*15q dup*): 31.86 ± 1.957, P = 0.9966<br>IL (WT): 33.67 ± 2.257 vs IL (*15q dup*): 31.43 ± 2.063, P = 0.8325<br>SC (WT): 31.17 ± 0.9866 vs SC (*15q dup*): 28.86 ± 1.432, P = 0.8164<br>MC (WT): 31.69 ± 1.309 vs MC (*15q dup*): 32.81 ± 1.460, P = 0.9840
**Fig. S2B (L V)**	The density of Synaptophysin-1-positive Puncta (Layer V)<br>Unit = Puncta/100 μm^2	n = 5 mice/ group	Two-way ANOVA, Sidak's post-hoc	Interaction (Region × Genotype): F(3,32) = 0.8860, P = 0.4588<br>Main effect (Region): F(3,32) = 1.063, P = 0.3783,<br>Main effect (Genotype): F(1,32) = 0.2057, P = 0.6532<br>Post-hoc:<br>PrL (WT): 31.43 ± 0.5051 vs PrL (*15q dup*): 32.07 ± 1.784, P = 0.9972<br>IL (WT): 32.47 ± 1.284 vs IL (*15q dup*): 32.94 ± 2.288 , P = 0.9992<br>SC (WT): 32.03 ± 2.022 vs SC (*15q dup*): 29.95 ± 1.494, P = 0.8187<br>MC (WT): 32.10 ± 0.9051 vs MC (*15q dup*): 35.05 ± 1.281, P = 0.5625

Table S5. Statistical details for the biochemical analysis and cluster quantification of axon initial segment (AIS) components in wild-type (WT) and *15q dup* m

Figure Panel	Description	Sample Size (n)	Statistical Test	Data (Median [25–75%] or Mean ± SEM) & Statistical Details
**Fig. S3A**	AnkG vs Pan-Nav (SC L II/III) >50 AIS/mouse	n = 4 mice/group	One-way ANOVA, Tukey's post-hoc	WT (Ankyrin-G): 24.57 (24.03-26.50) µm<br>WT (Pan-Nav): 25.83 (25.11-27.11) µm<br>*15q dup* (Ankyrin-G): 26.73 (26.47-28.56) µm<br>*15q dup* (Pan-Nav): 28.20 (27.03-28.57) µm<br>**ANOVA: F(3, 12) = 4.898, P = 0.0190 (*), η2 = 0.5505**<br>**Post-hoc:** WT(Ankyrin-G) vs WT(Pan-Nav) (P =0.2672), *15q dup* (Ankyrin-G) vs *15q dup* (Pan-Nav) (P =0.4276), **WT(Ankyrin-G) vs *15q dup* (Ankyrin-G) (P =0.0194), WT(Pan-Nav) vs *15q dup* (Pan-Nav), P =0.0387(*)**
**Fig. S3B**	AnkG vs Pan-Nav (PrL L V) >50 AIS/mouse	n = 4 mice/group	One-way ANOVA, Tukey's post-hoc	WT (Ankyrin-G): 28.01 (26.82-28.38) µm<br>WT (Pan-Nav): 28.68 (27.72-29.73) µm<br>*15q dup* (Ankyrin-G): 24.43 (23.65-24.92) µm<br>*15q dup* (Pan-Nav): 25.25 (23.86-25.31) µm<br>**ANOVA: F(3, 12) = 22.96, P < 0.0001 (****), η2 = 0.8516**<br>Post-hoc: WT(Ankyrin-G) vs WT(Pan-Nav) (P =0.4513), *15q dup* (Ankyrin-G) vs *15q dup* (Pan-Nav) (P =0.8778), **WT(Ankyrin-G) vs *15q dup* (Ankyrin-G) (P =0.0009***)**, **WT(Pan-Nav) vs *15q dup* (Pan-Nav), P =0.0003(***)**
**Fig. S3C**	Western Blot (Protein Ratio)	n = 6 mice/group	Two-tailed unpaired Student's t-test	Ankyrin-G (480 kDa): t=1.424, df=8, WT: 0.3741 ± 0.01913 vs *15q dup*: 0.4102 ± 0.01655, P = 0.1923 (ns)<br>Ankyrin-G (270 kDa): t=0.06971, df=8, WT: 0.3524 ± 0.03773 vs *15q dup*: 0.3491 ± 0.02912, P = 0.9461 (ns)<br>**Ankyrin-G (190 kDa): t=2.492, df=8, WT: 0.3640 ± 0.01273 vs *15q dup*: 0.2857 ± 0.02874, P = 0.0374 (*)**<br>Pan-Nav: t=1.767, df=8, WT: 1.155 ± 0.05281 vs *15q dup*: 1.015 ± 0.05873, P = 0.1153 (ns)
**Fig. S3D**	Real-time PCR (Transcript Ratio)	n = 6 mice/group	Two-tailed unpaired Student's t-test	Ankyrin-G: t=0.7052,df=10, WT: 0.03616 ± 0.001091 vs *15q dup*: 0.03890 ± 0.003732, P = 0.4968 (ns)<br>β4-spectrin: t=0.6587, df=10, WT: 0.001266 ± 0.0001615 vs *15q dup*: 0.001125 ± 0.0001416, P = 0.5249 (ns)<br>Nav1.2: t=0.9725, df=10, WT: 0.01889 ± 0.005055 vs *15q dup*: 0.02668 ± 0.006220, P = 0.3538 (ns)<br>Nav1.6: t=1.183, df=10, WT: 0.09373 ± 0.007036 vs *15q dup*: 0.1080 ± 0.009804, P = 0.2641 (ns)

Table S6. Statistical details of Ankyrin-G and Pan-Nav channel cluster lengths in layer II/III pyramidal neurons of the PrL, IL, and MC in wild-type (WT) and *15q dup* mice.

Figure Panel	Description	Sample Size (n)	Statistical Test	Data (Median [25–75%] & Statistical Details
**Fig. S4A**	AnkG vs Pan-Nav (PrL L II/III) >50 AIS/mouse	n = 4 mice/group	One-way ANOVA, Tukey's post-hoc	WT (Ankyrin-G): 27.71 (26.32-30.50) µm<br>WT (Pan-Nav): 27.78 (26.64-28.67) µm<br>*15q dup* (Ankyrin-G): 27.95 (27.52-29.45) µm<br>*15q dup* (Pan-Nav): 25.89 (25.38-27.50) µm<br>ANOVA: $F(3, 12) = 1.580$ $P = 0.2455$ (ns), $\eta 2 = 0.2832$<br>Post-hoc: WT(Ankyrin-G) vs WT(Pan-Nav) (P =0.9676), *15q dup* (Ankyrin-G) vs *15q dup* (Pan-Nav) (P =0.2629), WT(Ankyrin-G) vs *15q dup* (Ankyrin-G) (P =0.9993), WT(Pan-Nav) vs *15q dup* (Pan-Nav) (P =0.5433)
**Fig. S4B**	AnkG vs Pan-Nav (IL L II/III) >50 AIS/mouse	n = 4 mice/group	One-way ANOVA, Tukey's post-hoc	WT (Ankyrin-G): 26.29 (24.89-26.66) µm<br>WT (Pan-Nav): 26.82 (25.54-27.26) µm<br>15q dup (Ankyrin-G): 27.39 (26.88-29.28) µm<br>15q dup (Pan-Nav): 26.99 (26.80-27.55) µm<br>ANOVA: $F(3, 12) = 2.670$, $P =0.0948$ (ns), $\eta 2 = 0.4003$<br>Post-hoc: WT(Ankyrin-G) vs WT(Pan-Nav) (P =0.8344), *15q dup* (Ankyrin-G) vs *15q dup* (Pan-Nav) (P =0.7243), WT(Ankyrin-G) vs *15q dup* (Ankyrin-G) (P =0.0783), WT(Pan-Nav) vs *15q dup* (Pan-Nav) (P =0.8461)
**Fig. S4C**	AnkG vs Pan-Nav (MC L II/III) >50 AIS/mouse	n = 4 mice/group	One-way ANOVA, Tukey's post-hoc	WT (Ankyrin-G): 29.06 (28.39-30.21) µm<br>WT (Pan-Nav): 27.87 (24.94-29.01) µm<br>*15q dup* (Ankyrin-G): 27.90 (25.32-29.37) µm<br>*15q dup* (Pan-Nav): 27.15 (27.06-29.30) µm<br>ANOVA: $F(3, 12) = 0.9635$, $P = 0.4415$ (ns), $\eta 2 = 0.1941$<br>Post-hoc: WT(Ankyrin-G) vs WT(Pan-Nav) (P =0.4374), *15q dup* (Ankyrin-G) vs *15q dup* (Pan-Nav) (P =0.9944), WT(Ankyrin-G) vs *15q dup* (Ankyrin-G) (P =0.5489), WT(Pan-Nav) vs *15q dup* (Pan-Nav) (P =0.9684)

Table S7. Statistical details of Ankyrin-G and Pan-Nav channel cluster lengths in layer V pyramidal neurons of the IL, SC and MC in wild-type (WT) and *15q dup* mice.

Figure Panel	Description	Sample Size (n)	Statistical Test	Data (Median [25–75%] & Statistical Details
**Fig. S5A**	AnkG vs Pan-Nav<br>(IL L V)<br>>50 AIS/mouse	n = 4<br>mice/group	One-way ANOVA,<br>Tukey's post-hoc	WT (Ankyrin-G: 25.80 (23.84-29.77) μm<br>WT (Pan-Nav): 26.80 (26.26-27.73) μm<br>*15q dup* (Ankyrin-G): 26.82 (22.89-27.33) μm<br>15q dup (Pan-Nav): 27.77 (26.58-29.45) μm<br>ANOVA: F(3, 12) = 0.6704, P = 0.5864 (ns), η2 = 0.1435<br>Post-hoc: WT(Ankyrin-G) vs WT(Pan-Nav) (P =0.9917), *15q dup* (Ankyrin-G) vs 15q dup (Pan-Nav) (P =0.5303), WT(Ankyrin-G) vs *15q dup* (Ankyrin-G) (P =0.9608), WT(Pan-Nav) vs *15q dup* (Pan-Nav) (P =0.9244)
**Fig. S5B**	AnkG vs Pan-Nav<br>(SC L V)<br>>50 AIS/mouse	n = 4<br>mice/group	One-way ANOVA,<br>Tukey's post-hoc	WT (Ankyrin-G): 25.86 (23.80-27.60) μm<br>WT (Pan-Nav): 27.61 (25.13-29.57) μm<br>15q dup (Ankyrin-G): 25.51 (24.10-27.60) μm<br>*15q dup* (Pan-Nav): 26.52 (25.38-27.61) μm<br>ANOVA: F(3, 12) = 0.7270, P =0.5552 (ns), η2 = 0.1538<br>Post-hoc: WT(Ankyrin-G) vs WT(Pan-Nav) (P =0.6011), 15q dup (Ankyrin-G) vs *15q dup* (Pan-Nav) (P =0.9375), WT(Ankyrin-G) vs *15q dup* (Ankyrin-G) (P >0.9999), WT(Pan-Nav) vs *15q dup* (Pan-Nav) (P =0.8953)
**Fig. S5C**	AnkG vs Pan-Nav<br>(MC L V)<br>>50 AIS/mouse	n = 4<br>mice/group	One-way ANOVA,<br>Tukey's post-hoc	WT (Ankyrin-G) :26.94 (24.85-29.91) μm<br>WT (Pan-Nav): 28.69 (27.36-29.10) μm<br>*15q dup* (Ankyrin-G): 27.79 (26.18-30.10) μm<br>*15q dup* (Pan-Nav): 26.62 (26.18-29.21) μm<br>ANOVA: F(3, 12) = 0.3067, P = 0.8201 (ns), η2 = 0.07122<br>Post-hoc: WT(Ankyrin-G) vs WT(Pan-Nav) (P =0.8459), *15q dup* (Ankyrin-G) vs *15q dup* (Pan-Nav) (P =0.9604), WT(Ankyrin-G) vs *15q dup* (Ankyrin-G) (P =0.9419), WT(Pan-Nav) vs *15q dup* (Pan-Nav) (P =0.8778)

Table S8. Statistical details of projection-specific axon initial segment (AIS) length in layer V pyramidal neurons of the prelimbic cortex in wild-type (WT) and *15q dup* mice.

Figure Panel	Description	Sample Size (n)	Statistical Test	Data (Median [25–75%] or Mean ± SEM) & Statistical Details
**Fig. 3C**	Projection-Specific AIS Length	n = 5 mice/group >50 AIS/mouse	Two-way ANOVA, Sidak's post-hoc	Interaction (Region × Genotype): F(4, 40) = 1.610, P = 0.1906.<br>Main effect (Region): F(4, 40) = 1.538, P = 0.2097<br>Main effect (Genotype): F(1, 40) = 11.68, P = 0.0015<br>Post-hoc (WT vs *15q dup*):<br>**NAcc:** 28.82 (28.08-29.48) µm (WT) vs 26.93 (25.31-27.38) µm (*15q dup*), **P = 0.0497 (*)**<br>CPVM: 27.75 (24.75-28.83) µm (WT) vs 26.88 (25.43-28.31) µm (*15q dup*), P = 0.9448 (ns)<br>**LHb:** 27.65 (26.70-28.23) µm (WT) vs 23.33 (23.05-25.50) µm (*15q dup*), **P = 0.0049 (**)**<br>VTA: 26.25 (23.87-28.93) µm (WT) vs 24.64 (23.90-28.78) µm (*15q dup*), P = 0.7478 (ns)<br>**DRN:** 27.70 (27.12-29.53)µm (WT) vs 25.81 (24.89-26.25) µm (*15q dup*), **P = 0.0301 (*)**
**Fig. 3D**	Projection-Specific AIS Length (Cumulative frequency distribution)	(n=5 mice)	Mann-Whitney U test	**NAcc:** U=0, 28.72 ± 0.4104 µm (WT) vs 26.46 ± 0.6579 µm (*15q dup*), **P = 0.0079 (**)**<br>CPVM: U=11, 6.95 ± 0.9346 µm (WT) vs 26.87 ± 0.8498 µm (*15q dup*), P = 0.8413 (ns)<br>**LHb:** U=1, 27.50 ± 0.4405 µm (WT) vs 24.08 ± 0.7046 µm (*15q dup*), **P = 0.0159 (*)**<br>VTA: U=12, 26.37 ± 1.206 µm (WT) vs 26.00 ± 1.370 µm (*15q dup*), P > 0.9999 (ns)<br>**DRN:** U=0, 28.20 ± 0.5685 µm (WT) vs 25.62 ± 0.3164 µm (*15q dup*), **P = 0.0079 (**)**

Table S9. Statistical details for the chemogenetic rescue of axon initial segment (AIS) shortening in DRN-projecting mPFC pyramidal neurons of *15q dup* mice.

Figure Panel	Description	Sample Size (n)	Statistical Test	Data (Median [25–75%] or Mean ± SEM) & Statistical Details	
**Fig 4E**	Rescue of DRN-Projecting Neurons (Saline vs DCZ)	n = 5 (Saline),<br>n = 5 (DCZ)<br>>50 AIS/mouse	Two-tailed unpaired Student's t-test	Saline: 24.44 (22.75-24.65) µm vs DCZ: 25.91 (25.70-27.06) µm<br>t(8)=4.120, **P = 0.0033 (**)**<br>F = 1.440, P = 0.7326, η2 = 0.6797	
**Fig 4F**	Rescue of DRN-Projecting Neurons (Cumulative frequency distribution)	n = 5 (Saline),<br>n = 5 (DCZ)	Mann-Whitney U test	**U=0, 23.85 ± 0.4546 µm vs DCZ: 26.28 ± 0.3789 µm, P = 0.0079 (**)**	
**Fig. 4H**	Confirmation of DCZ Specificity (All Genotypes)	n = 5 mice/group<br>>50 AIS/mouse	One-way ANOVA, Tukey's post-hoc	WT: 26.08 (25.61-27.38) µm<br>*15q dup*: 24.02 (22.61-24.83) µm<br>*Rbp4-Cre*: 27.29 (25.66-29.01) µm<br>*15q dup;Rbp4-Cre*: 25.91 (25.70-27.06) µm<br>**ANOVA:** F(3, 16) = 7.126, **P = 0.0030(**)**, η2 = 0.5719	Post-hoc:<br>**WT vs *15q dup*: P = 0.0225 (*)**<br>WT vs *WT;Rbp4-Cre*: P = 0.6728 (ns)<br>WT vs *15q Dup-Rbp4-*Cre: P = 0.9986 (ns)<br>***15q dup* vs WT;*Rbp4-Cre*: P = 0.0022 (**)**<br>***15q dup* vs *15q dup;Rbp4-Cre*: P = 0.0307 (*)**<br>*WT;Rbp4-Cre* vs *15q dup;Rbp4-Cre*: P = 0.5797 (ns)
**Fig. 4I**	Confirmation of DCZ Specificity (Cumulative frequency distribution)	n = 5 mice/group	One-way ANOVA, Tukey's post-hoc	**ANOVA:** F(3, 16) = 7.126, **P = 0.0030(**)**, η2 = 0.5719<br>WT: 26.41 ± 0.4066 µm,*15q dup:* 23.78 ± 0.5676 µm,<br>*Rbp4-Cre*: 27.33 ± 0.8164 µm, *15q dup;Rbp4-Cre*:<br>26.28 ± 0.3789 µm	Post-hoc:<br>**WT vs *15q dup*: P = 0.0225 (*)**<br>WT vs *WT;Rbp4-Cre*: P = 0.6728 (ns)<br>WT vs *15q Dup-Rbp4-*Cre: P = 0.9986 (ns)<br>***15q dup* vs WT;*Rbp4-Cre*: P = 0.0022 (**)**<br>***15q dup* vs *15q dup;Rbp4-Cre*: P = 0.0307 (*)**<br>*WT;Rbp4-Cre* vs *15q dup;Rbp4-Cre*: P = 0.5797 (ns)
**Fig. 4J, K**	DREADD-Expressing (mCherry+) vs Non-Expressing (mCherry-) Cells	n = 5 mice/group<br>>50 AIS/mouse	One-way ANOVA, Tukey's post-hoc	*Rbp4-Cre* (mCherry+): 27.29 (25.66-29.01) µm<br>*Rbp4-Cre* (mCherry–): 26.64 (26.48-27.52) µm<br>*15q dup;Rbp4-Cre* (mCherry+): 25.91 (25.70-27.06) µm<br>*15q dup;Rbp4-Cre* (mCherry–): 24.25 (23.89-24.74) µm<br>**ANOVA:** F(3, 16) = 7.707, **P = 0.0021 (**)**, η2 = 0.5910<br>*Rbp4-Cre* (mCherry+): 27.33 ± 0.8164 µm,<br>*Rbp4-Cre* (mCherry–): 26.92 ± 0.2563 µm,<br>*15q dup;Rbp4-Cre* (mCherry+): 26.28 ± 0.3789 µm,<br>*15q dup;Rbp4-Cre* (mCherry–): 24.30 ± 0.2450 µm	Post-hoc:<br>*WT; Rbp4-Cre* (mCherry+) vs WT; *Rbp4-Cre* (mCherry–): P = 0.9343 (ns)<br>*WT; Rbp4-Cre* (mCherry+) vs 15*q dup*; *Rbp4-Cre* (mCherry–): P = 0.4473(ns)<br>***WT; Rbp4-Cre* (mCherry+) vs 15*q dup*; *Rbp4-Cre* (mCherry+): P = 0.0022 (**)**<br>*WT; Rbp4-Cre* (mCherry-) vs 15*q dup*; *Rbp4-Cre* (mCherry+): P = 0.7864(ns)<br>***WT; Rbp4-Cre* (mCherry-) vs 15*q dup*; *Rbp4-Cre* (mCherry–): P = 0.0071(**)**<br>***15q dup;Rbp4-Cre* (mCherry+) vs *15q dup;Rbp4-Cre* (mCherry–): P = 0.0463 (*)**

Table S10. Statistical details for the chemogenetic rescue of behavioral deficits in *15q dup* mice via activation of DRN-projecting mPFC pyramidal neurons.

Figure Panel	Description	Sample Size (n)	Statistical Test	Data (Mean ± SEM) & Statistical Details	
**Fig. 5C (Preference)**	3-CT (Social Preference, Time)	n = 11 mice/group	Two-way ANOVA, Sidak's post-hoc	**Interaction (Stimulation × Genotype): F(3, 84) = 10.63, P < 0.0001.**<br>**Main effect (Stimulation): F(1, 84) = 55.87, P < 0.0001**<br>**Main effect (Genotype): F(3, 84) = 2.859, P = 0.0418**<br>**WT (S1 vs NS):** 103.43 ± 9.47 vs 45.07 ± 5.03, **P < 0.0001 (****)**<br>*15q dup (S1 vs NS): 53.58 ± 9.35 vs 66.82 ± 9.37, P = 0.9998 (ns)*<br>**WT;*Rbp4-Cre* (S1 vs NS):** 119.62 ± 10.95 vs 47.99 ± 4.83, **P < 0.0001 (****)**<br>***15q dup;Rbp4-Cre (S1 vs NS): 96.34 ± 7.85 vs 40.39 ± 6.29, P = 0.0002 (***)***	
**Fig. 5C (Novelty)**	3-CT (Social Novelty, Time)	n = 11 mice/group	Two-way ANOVA, Sidak's post-hoc	**Interaction (Stimulation × Genotype): F(3, 84) = 6.951, P = 0.0003.**<br>**Main effect (Stimulation): F(1, 84) = 17.35, P < 0.0001**<br>Main effect (Genotype): F(3, 84) = 0.1535, P = 0.9272<br>**WT (S2 vs S1):** 74.43 ± 6.90 vs 37.50 ± 5.36, **P = 0.0351 (*)**<br>*15q dup (S2 vs S1): 48.04 ± 9.41 vs 70.64 ± 7.99, P = 0.8430 (ns)*<br>**WT;*Rbp4-Cre* (S2 vs S1):** 83.58 ± 12.74 vs 39.25 ± 3.21, **P = 0.0114 (*)**<br>***15q dup;Rbp4-Cre (S2 vs S1): 78.37 ± 10.70 vs 38.67 ± 7.81, P = 0.0394(*)***	
**Fig. 5C (Pref. Index)**	3-CT (Social Preference, Index)	n = 11 mice/group	One-way ANOVA, Tukey's post-hoc	**ANOVA: F(3, 42) = 10.11, P < 0.0001 (****), η^2 = 0.4194**<br>WT: 0.3776 ± 0.07061<br>*15q dup: -0.1072 ± 0.1036*<br>WT;*Rbp4-Cre*: 0.4086 ± 0.07303<br>*15q dup;Rbp4-Cre: 0.4256 ± 0.06633*	Post-hoc:<br>***WT vs 15q dup: P = 0.0004 (**).***<br>WT vs WT;Rbp4-Cre: P = 0.9921 (ns).<br>*WT vs 15q dup;RBP4-Cre: P = 0.9717 (ns).*<br>***15q dup vs WT;Rbp4-Cre: P = 0.0003 (**).***<br>*15q dup vs 15q dup;RBP4-Cre: P = 0.0002 (***).*<br>*WT;Rbp4-Cre vs 15q dup;RBP4-Cre: P = 0.9988 (ns).*
**Fig. 5C (Nov. Index)**	3-CT (Social Novelty, Index)	n = 11 mice/group	One-way ANOVA, Tukey's post-hoc	**ANOVA: F(3, 42) = 8.792, P = 0.0001 (***), η^2 = 0.3857**<br>**WT:** 0.3403 ± 0.06526<br>*15q dup: -0.2208 ± 0.1035*<br>***WT;Rbp4-Cre*:** 0.2938 ± 0.08909<br>*15q dup;Rbp4-Cre: 0.3462 ± 0.1096*	Post-hoc:<br>***WT vs 15q dup: P = 0.0004 (**).***<br>WT vs WT;Rbp4-Cre: P = 0.9830 (ns).<br>*WT vs 15q dup;RBP4-Cre: P > 0.9999 (ns).*<br>***15q dup vs WT;Rbp4-Cre: P = 0.0019 (**).***<br>***15q dup vs 15q dup;RBP4-Cre: P = 0.0006 (**).***<br>*WT;Rbp4-Cre vs 15q dup;RBP4-Cre: P = 0.9786 (ns).*
**Fig. 5E**	Marble Burying Test (MBT)	n = 11 mice/group	One-way ANOVA, Tukey's post-hoc	**ANOVA: F(3, 42) = 9.151, P < 0.0001 (****), η^2 = 0.3953**<br>**WT:** 7.846 ± 1.159<br>*15q dup: 15.55 ± 1.048*<br>**WT;*Rbp4-Cre*:** 9.455 ± 1.115<br>*15q dup;Rbp4-Cre: 8.182 ± 1.354*	Post-hoc:<br>***WT vs 15q dup: P = 0.0002 (**).***<br>WT vs WT;Rbp4-Cre: P = 0.7593 (ns).<br>*WT vs 15q dup;Rbp4-Cre: P = 0.9969 (ns).*<br>***15q dup vs WT;Rbp4-Cre: P = 0.0047 (**).***<br>***15q dup vs 15q dup;Rbp4-Cre: P = 0.0005 (**).***<br>*WT;Rbp4-Cre vs 15q dup;Rbp4-Cre: P = 0.8770 (ns).*

Supplementary Materials

REAGENT or RESOURCE	SOURCE	IDENTIFIER
Antibodies		
Anti-Ankyrin-G (Mouse; N106/36)<br>1:1000 (immunostaining)	UC Davis/NIH NeuroMab Facility	Cat# N106/36, RRID:AB_2877524
Anti-Ankyrin-G (Guinea pig)<br>1:500 (immunostaining), 1:1000 (Western blot),	Synaptic Systems	Cat# 386 004, RRID:AB_2725774
Anti-Pan-sodium channel antibody (Mouse; K58/35)<br>1:1000 (immunostaining), 1:1000 (Western blot),	Sigma-Aldrich	Cat# S8809, RRID:AB_477552
Anti- Ctip2 antibody (Rat; 25B6)<br>1:2000 (immunostaining)	Abcam	Cat# ab18465, RRID:AB_2064130
Anti-Cux1 antibody (Rabbit)<br>1:250 (immunostaining)	Proteintech	Cat# 11733-1-AP, RRID:AB_2086995
Anti-CTB antibody (Goat)<br>1:5000 (immunostaining)	List Biological	Cat# 703, RRID:AB_10013220
Anti-c-Fos Antibody (Rabbit)<br>1:1000 (immunostaining)	Cell Signaling Technology	Cat# 4384, RRID:AB_2106617
Anti-Synaptophysin1 (p38-1) (Guinea pig)<br>1:500 (immunostaining)	Synaptic Systems	Cat# 101 004 RRID:AB_1210382
Anti-beta actin (C-4) (Mouse)<br>1:3000 (Western blot)	Santa Cruz Biotechnology	Cat# sc-47778, RRID:AB_626632
Anti-EB3 (7) (Mouse)<br>1:200 (immunostaining)	Santa Cruz Biotechnology	Cat# sc-136405, RRID:AB_10707846
Anti-mCherry antibody (Chick)<br>1:4000 (immunostaining)	EnCor Biotechnology	Cat# CPCA-mCherry, RRID:AB_2572308
Donkey anti-Mouse IgG(H+L) Alexa Fluor 647 plus<br>1:1000 (immunostaining)	Thermo Fisher Scientific	Cat# A32787TR, RRID:AB_2866494
Donkey anti-Rat IgG(H+L) Alexa Fluor 405 plus<br>1:500 (immunostaining)	Thermo Fisher Scientific	Cat# A48268, RRID:AB_2890549
Cy3-AffiniPure Donkey Anti-Rat IgG (H+L) (min X Bov,Ck,Gt,GP,Sy Hms,Hrs,Hu,Ms,Rb,Shp Sr Prot)<br>1:1000 (immunostaining)	Jackson ImmunoResearch Labs	Cat# 712-165-153, RRID:AB_2340667)
Donkey anti-Rabbit IgG (H+L)Alexa Fluor 405 plus<br>1:500 (immunostaining)	Thermo Fisher Scientific	Cat# A48258, RRID:AB_2890547
Donkey anti-Rabbit IgG (H+L) Alexa Fluor 555 plus<br>1:1000 (immunostaining)	Thermo Fisher Scientific	Cat# A32794, RRID:AB_2762834)
Cy3-AffiniPure Fab Fragment Donkey Anti-Goat IgG (H+L)<br>1:1000 (immunostaining)	(Jackson ImmunoResearch Labs	Cat# 705-167-003, RRID:AB_2340414)
Cy3-AffiniPure Donkey Anti-Chicken IgY (IgG) (H+L) (min X Bov,Gt,GP,Sy Hms,Hrs,Hu,Ms,Rb,Rat,Shp Sr Prot)<br>1:1000 (immunostaining)	Jackson ImmunoResearch Labs	Cat# 703-165-155, RRID:AB_2340363)

Peroxidase-AffiniPure Goat Anti-Mouse IgG (H + L)<br>1:10000 (Western blot)	Jackson ImmunoResearch Labs	Cat# 115-035-003, RRID:AB_10015289)
Peroxidase-AffiniPure Goat Anti-Rabbit IgG (H+L)<br>1:10000 (Western blot)	Jackson ImmunoResearch Labs	Cat# 111-035-003, RRID:AB_2313567)
Peroxidase-AffiniPure Donkey Anti-Guinea Pig IgG (H+L) (min X Bov,Ck,Gt,Sy Hms,Hrs,Hu,Ms,Rb,Rat,Shp Sr Prot)<br>1:10000 (Western blot)	Jackson ImmunoResearch Labs	Cat# 706-035-148, RRID:AB_2340447
Bacterial and virus strains		
E. coli DH5α Competent Cells	Takara Bio	Cat # 9057
AAV9-nEF Con/Fon DREADD Gq-mCherry	This study	N/A
AAV-retrograde-EF1a-Flpo	This study	Cat # 55637-AAVrg
Chemicals, peptides, and recombinant proteins		
Paraformaldehyde	Sigma-Aldrich	Cat# 158127
Glyoxal	Sigma-Aldrich	Cat# 128465
Normal donkey serum	Chemicon/Merck	Cat# S30-100ML
VECTASHIELD PLUS Antifade Mounting Medium	Vector Laboratories	Cat# H-1900
Radioimmunoprecipitation assay (RIPA) buffer	Wako pure/FUJIFILM	Cat# 182-02451
Protease Inhibitor Cocktail Set III	Wako pure/FUJIFILM	Cat# 163-26061
bicinchoninic acid (BCA) assay<br>(Pierc BCA Protein Assay Kits)	Thermo Fisher Scientific	Cat# 23227
NeuroTrace® Green 500/525 Fluorescent Nissl Stain	Thermo Fisher Scientific	Cat# N21480
Cellstain® DAPI solution	Dojindo	Cat# D523
Blocking One	Nacalai Tesque	Cat# 03953-66
ECL Western Blotting Detection Reagents	Amersham/Cytiva,	Cat# RPN2109
Isogen II reagent	Nippon Gene	Cat# 311-07361
ReverTra Ace qPCR RT Master Mix with gDNA Remover	Toyobo	Cat# FSQ-301
THUNDERBIRD NEXT SYBR qPCR Mix	Toyobo	Cat# QPX-201
Dulbecco's modified Eagle's medium	Wako pure/FUJIFILM	Cat# 044-29765
Fetal bovine serum	Hyclone/Cytiva	Cat# SH30910.03
Polyethyleneimine Max	Polysciences, Inc	Cat# 24765
PEG (polyethylene glycol)	Sigma-Aldrich	Cat# P5413
Benzonase Nuclease	Merck Millipore	Cat# 70746
OptiPrep	Serumwerk Bernburg AG	Cat# 1893
cholera toxin subunit B	List Biological	Cat# 104
deschloroclozapine (DCZ)	MedChemExpress	Cat# HY-42110
Experimental models: Cell lines		
HEK293T	Riken BRC	RRID:RCB2202
Experimental models: Organisms/strains		
C57BL/6J	Jackson Laboratory	RRID:IMSR_JAX:000664
B6.129S7-Dp(7Herc2-Mkrn3)1Taku	Nakatani et al[22]	RRID:IMSR_RBRC05954

B6.FVB(Cg)-Tg(*Rbp4-Cre*)KL100Gsat/Mmucd	Mutant Mouse Resource & Research Centers	(RRID: MMRRC_037128-UCD)
Oligonucleotides		
Genotyping for *15q dup* mice:<br>hprt-intron2-F1: AGAGGAGGGCCTTACTAATTACTTA;<br>hprt-intron2-R2: ATATGTACTTTTGCATATAGTATAC;<br>olMR0015: AAATGTTGCTTGTCTGGTG;<br>olMR0016: GTCAGTCGAGTGCACAGTTT	Nakatani et al [22]	N/A
Genotyping for *RBP4-Cre* mice:<br>Forward, GGGCGGCCTCGGTCCTC;<br>Reverse, CCCCAGAAATGCCAGATTACGTAT	Mutant Mouse Resource & Research Centers	N/A
Ankyrin-G: Forward, GTCTTGCTTCAGAACAACGCCTC;<br>Reverse, TTTCCTCCGTCACGACCTTCAG	This study	N/A
Beta4-spectrin (Sptbn4): Forward, ACTACTGGACCAGCTTGTGCTG;<br>Reverse, GAGCCATCTCTTGTGCAGCTTG	This study	N/A
Sodium channel 1.2 voltage-gated, type II, alpha (Scn2a): Forward, ATTTTCGGCTCATTCTTCACACT;<br>Reverse, GGGCGAGGTATCGGTTTTTGT	This study	N/A
Sodium channel 1.6 voltage-gated, type VIII, alpha (Scn8a): Forward, GCAAGCTCAAGAAACCACCC;<br>Reverse, CCGTAGATGAAAGGCAAACTCT	This study	N/A
Actb (actin, beta): Forward, TTTGCAGCTCCTTCGTTGC;<br>Reverse, ACGATGGAGGGGAATACAGC	This study	N/A
AAV virus Titer check Primer (AAV2 ITR):<br>Fwd ITR primer, 5'-GGAACCCCTAGTGATGGAGTT;<br>Rev ITR primer, 5'-CGGCCTCAGTGAGCGA	This study	Addgene protocol https://www.addgene.org/protocols/aav-titration-qpcr-using-sybr-green-technology/
Recombinant DNA		
pAAV-nEF Con/Fon DREADD Gq-mCherry	Karl Deisseroth	RRID: Addgene_183532
pUCmini-iCAP-AAV9-X1.1	Viviana Gradinaru	RRID: Addgene_196836
pAAV-EF1a-Flpo	Karl Deisseroth	RRID: Addgene_55637
pHelper	Agilent Technologies	Cat# 240071
Software and algorithms		
Clampfit 10.7.0 software	Molecular Devices	RRID:SCR_011323
ImageJ	National Institutes of Health	RRID:SCR_014210
GraphPad Prism 9	GraphPad Software	RRID:SCR_002798
ANY-maze v7.34	Stoelting Co.	RRID:SCR_014289
Other		

Sliding microtome HM430	Thermo Fisher Scientific	Cat# MIC910010
FV-1000D and FV-3000D confocal microscopes	Evident / Olympus	https://evidentscientific.com/ja/products/obsolete/fv3000
Vibratome 7000smz-2	Campden	https://campdeninstruments.com/products/7000smz-2-vibrating-microtome
BX51WI microscope	Evident / Olympus	https://evidentscientific.com/ja/products/upright/bxwi
Axon Axopatch 200B Microelectrode Amplifier	Molecular Devices	https://www.moleculardevices.com/sites/default/files/en/assets/data-sheets/dd/cns/axon-axopatch-200b-microelectrode-amplifier.pdf
E-T520L e-PAGEL mini gels（5～20%）	ATTO Corporation	Cat# 2331830
Immobilon-P polyvinylidene fluoride (PVDF) membranes	Merck Millipore,	Cat# IPVH00005
BioMasher II	Nippi/ FUJIFILM	Cat# 383-21581
Thermal Cycler Dice Real-Time System II TP900	Takara Bio	N/A
ultrasonic homogenizer SONIFIER 450 Advanced	Branson Ultrasonics Corporation	N/A
ImageQuant 800 system	Amersham/Cytiva,	https://www.cytivalifesciences.co.jp/catalog/45507.html
L-60 ultracentrifuge and 60Ti rotor	Beckman Coulter	N/A
Vivaspin 20 100000 MWCO PES	Sartrius	Cat# VS2042
The 3-Chamber Test apparatus	SHINFACTORY, Inc.	Cat# M3C-10
