# Supplementary material for: Restoration of axon initial segment plasticity via chemogenetic activation rescues autism-related behaviors
Source: Cell Death Dis. 2026 May 19;17(1):634. doi: 10.1038/s41419-026-08873-0 (PMC13357743; doi:10.1038/s41419-026-08873-0)
Supplement: Supplementary file 2 — Original Data [file 41419_2026_8873_MOESM2_ESM.pdf]

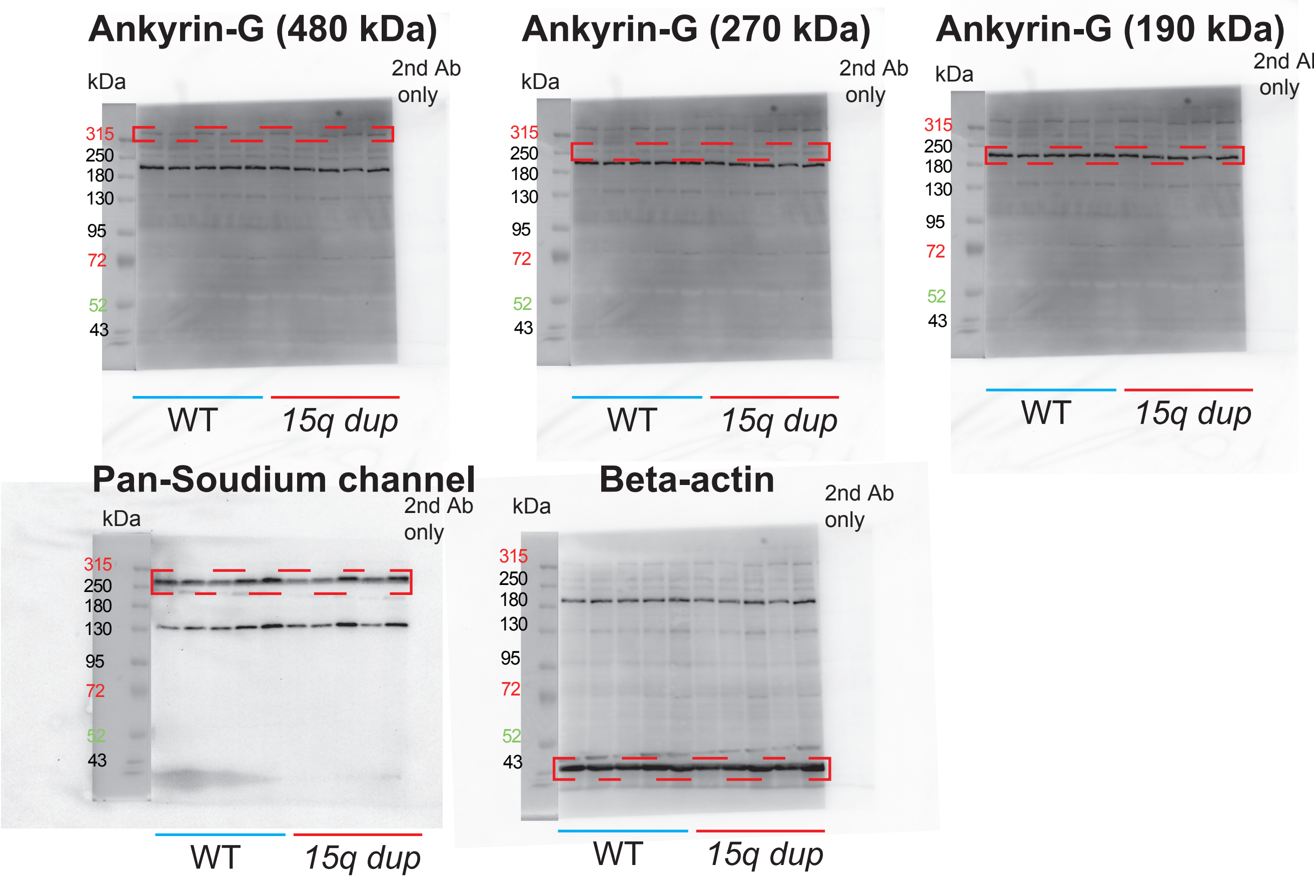

Figure S6. Full uncropped immunoblots related to Figure S3C

Full, uncropped western blot images corresponding to the quantified data presented in Figure S3C. Blots were probed for Ankyrin-G (480, 270, and 190 kDa), Pan-voltage-gated sodium (Pan-NaV) channels, and β-actin (loading control). Lanes labeled "2nd Ab only" are negative controls in which the primary antibody was omitted, confirming the specificity of the secondary antibody.
